# Adherence to Mediterranean Diet and Maternal Lifestyle during Pregnancy: Island–Mainland Differentiation in the CRIBS Birth Cohort

**DOI:** 10.3390/nu12082179

**Published:** 2020-07-22

**Authors:** Dubravka Havaš Auguštin, Jelena Šarac, Mario Lovrić, Jelena Živković, Olga Malev, Nives Fuchs, Natalija Novokmet, Mirjana Turkalj, Saša Missoni

**Affiliations:** 1 Institute for Anthropological Research, 10000 Zagreb, Croatia; nives.fuchs@inantro.hr (N.F.); natalija.novokmet@inantro.hr (N.N.); sasa.missoni@inantro.hr (S.M.); 2 Centre for Applied Bioanthropology, Institute for Anthropological Research, 10000 Zagreb, Croatia; 3 Department of Translational Medicine, Srebrnjak Children’s Hospital, 10000 Zagreb, Croatia; mlovric@know-center.at (M.L.); zivkovic@bolnica-srebrnjak.hr (J.Ž.); olga.malev@bolnica-srebrnjak.hr (O.M.); 4 Know-Center, 8010 Graz, Austria; 5 Division of Zoology, Department of Biology, Faculty of Science, University of Zagreb, 10000 Zagreb, Croatia; 6 Department of Pulmonology and Allergology for Infants and Young Children, Srebrnjak Children’s Hospital, 10000 Zagreb, Croatia; turkalj@bolnica-srebrnjak.hr; 7 Department of Nursing, Catholic University of Croatia, 10000 Zagreb, Croatia; 8 School of Medicine, “J. J. Strossmayer” University, 31000 Osijek, Croatia

**Keywords:** Mediterranean diet, Dalmatian islands, pregnancy, CRIBS cohort, socioeconomic factors, lifestyle, machine learning, random forest, nutrition

## Abstract

Maternal nutrition and lifestyle in pregnancy are important modifiable factors for both maternal and offspring’s health. Although the Mediterranean diet has beneficial effects on health, recent studies have shown low adherence in Europe. This study aimed to assess the Mediterranean diet adherence in 266 pregnant women from Dalmatia, Croatia and to investigate their lifestyle habits and regional differences. Adherence to the Mediterranean diet was assessed through two Mediterranean diet scores. Differences in maternal characteristics (diet, education, income, parity, smoking, pre-pregnancy body mass index (BMI), physical activity, contraception) with regards to location and dietary habits were analyzed using the non-parametric Mann–Whitney U test. The machine learning approach was used to reveal other potential non-linear relationships. The results showed that adherence to the Mediterranean diet was low to moderate among the pregnant women in this study, with no significant mainland–island differences. The highest adherence was observed among wealthier women with generally healthier lifestyle choices. The most significant mainland–island differences were observed for lifestyle and socioeconomic factors (income, education, physical activity). The machine learning approach confirmed the findings of the conventional statistical method. We can conclude that adverse socioeconomic and lifestyle conditions were more pronounced in the island population, which, together with the observed non-Mediterranean dietary pattern, calls for more effective intervention strategies.

## 1. Introduction

The Mediterranean dietary pattern has been proved to have a protective effect against obesity, metabolic syndrome (MetS), cardiovascular diseases, and cancer due to a high consumption of antioxidants and a low intake of saturated fats [1,2]. It is characterized by a high intake of whole foods, fruits, vegetables, whole grain cereals, legumes, fish, and nuts and extra-virgin olive oil, low-to-moderate consumption of dairy products, smaller amounts of white meat and red wine, and a minimal consumption of processed food, red meat, animal fats, and sugar [3]. In this context, the beneficial effects of the Mediterranean diet on the mother’s health during gestation, as well as the importance of maternal health for the child later in life has been well established [4,5,6,7,8,9,10]. However, the influence of the maternal diet is also interlinked with many other lifestyle factors, including smoking, body mass index (BMI), education, age, urban versus rural residency etc., [11]. Although healthy food patterns and lifestyle should be priorities during pregnancy, the results of two recent studies based on birth cohort have indicated that the general maternal adherence to the Mediterranean diet in Europe is low and does not meet the recommendations [12]. However, it increases with age, social class, education, and generally healthy lifestyle [11,13]. Furthermore, smoking before pregnancy and a higher BMI in early pregnancy were reported as related to one another and associated with poor eating behavior [11]. Numerous studies undertaken on general population in the last decade point out that both dietary and lifestyle habits of the Mediterranean population are gradually shifting from the traditional patterns to the ”Westernization” of societies, which is especially apparent in the younger generations [14]. The results of a south Italian cohort reported that the rate of high adherence to the Mediterranean diet dropped from about 31% to 18% between 2005 and 2010 [15].

Poor compliance with the Mediterranean diet and unhealthy lifestyle have also been observed in the Croatian rural, Mediterranean region of Dalmatia, although the national food-based dietary guidelines for Croatia from 2002 follow the Mediterranean dietary pattern [16]. The traditional diet, which includes home-prepared unprocessed food made of homegrown fruits and vegetables, and dairy products from free-range goats and sheeps, has until recently been dominant in smaller rural communities in Croatia, especially on the islands. However, the traditional diet and lifestyle of islanders is changing under the influence of modernization from 1990s onwards. These changes are characterized by an increased consumption of red meat, poultry, milk, dairy products, sugar, and industrial, processed products and a decreased consumption of fish, fruits, and vegetables [17,18]. The observed negative trend is also accompanied by a drastically reduced level of physical activity and it has been singled out as a strong trigger for the development of diverse pathogenic changes i.e., general overweight and obesity trend, the occurrence of metabolic syndrome, diabetes mellitus, and coronary heart diseases [19,20].

Although preliminary results of the Croatian Islands’ Birth Cohort Study (CRIBS) reported general adherence of pregnant women to the Mediterranean dietary pattern in a small sample of participants [21], a comprehensive study on dietary habits in Croatian pregnant women (especially on the islands) is still lacking. Other CRIBS results also confirmed the differences between Croatian island and mainland population, suggesting adverse socioeconomic and lifestyle conditions in the insular population [22,23]. This is in line with previous reports which indicated a greater burden of cardiovascular disease (CVD) risk factors in the population from the rural areas of Croatia [24,25].

**Figure 1:**
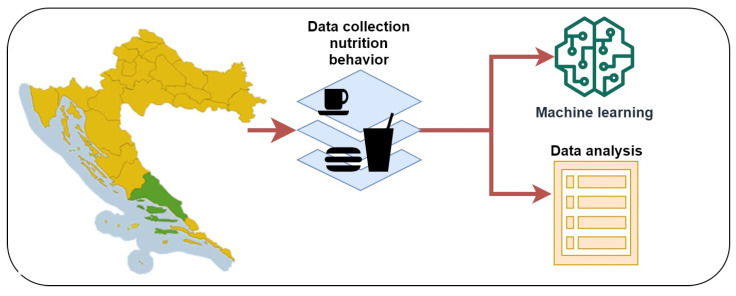
Graphical abstract of the present study.

The aim of this study is to extend the current knowledge on dietary and lifestyle habits among pregnant women in Dalmatia, Croatia, highlighting the major mainland–island differences. Croatian island population has been in the focus of epidemiological and nutritional studies for decades and a more traditional, Mediterranean background and lifestyle patterns would be expected due to their geographic confinement. However, current globalization trends have influenced the aforementioned traditional communities substantially and this seems to be reflected in the population of pregnant women as well. Additionally, studies on the Mediterranean diet adherence in island populations are generally scarce, especially in pregnant women and no specific nutritional guidelines have been drawn up for this sensitive subpopulation in Croatia. The specific objectives of this study are: (i) to investigate the level of maternal adherence to the Mediterranean diet in the Croatian Islands’ Birth Cohort Study (CRIBS), (ii) to correlate the dietary habits with different maternal socioeconomic and lifestyle characteristics and environmental exposures (education, income, smoking, pre-pregnancy BMI, parity etc.), (iii) to detect island–mainland differences in food choices, the Mediterranean diet adherence, and other socioeconomic and lifestyle characteristics using two different methodological approaches (conventional statistics and machine learning) (Figure 1).

## 2. Materials and Methods

The data used in this study are a part of the “Croatian Islands Birth Cohort Study (CRIBS)”. CRIBS was the first Croatian birth cohort study designed to prospectively follow a sample of 500 pregnant women and their children up to two years of age in the population from the Croatian Dalmatian islands (Hvar and Brač) and the mainland population (city of Split). One of the aims of this pilot study was to observe the differences between the Croatian island and mainland sub-population with regards to the risk factors (i.e., biological, environmental, and behavioral) for metabolic syndrome (MetS). The enrolment of pregnant woman in the CRIBS study and three follow-ups during pregnancy (12th–14th week of gestation, 22nd–26th week of gestation, 30th–32nd week of gestation) were carried out in the gynecology practices in Split and on the islands of Brač and Hvar where they received prenatal care. The inclusion criteria and recruitment were described previously in Perinić Lewis et al. [22]. All CRIBS participants signed an informed consent for the study participation and record linkage prior to their inclusion in the study and they all gave birth at the University Hospital Centre in Split. The research was performed in accordance with the Declaration of Helsinki. The Ethical Committee Approval for the CRIBS study was obtained from the Institute for Anthropological Research (Zagreb, Croatia) and Srebrnjak Children’s Hospital (SCH), Zagreb Croatia. Collection of all data and anthropometric measurements was performed in accordance with the relevant guidelines and regulations.

**Figure 2:**
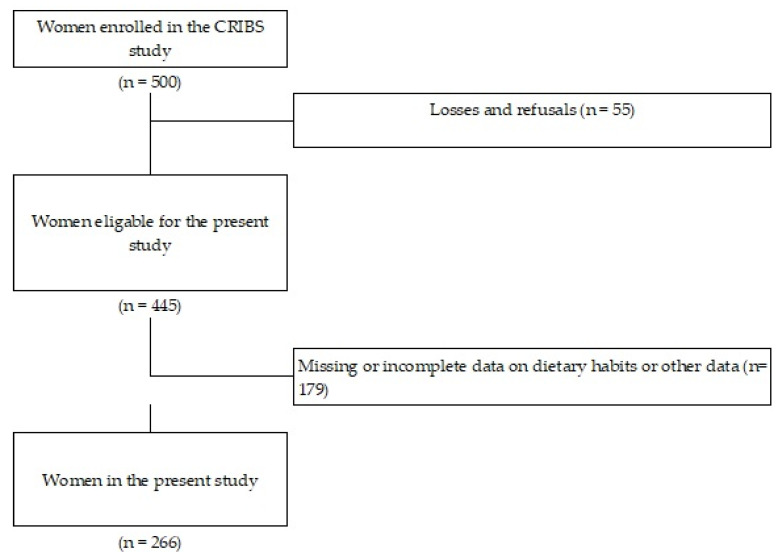
Study flow chart.

266 pregnant women from the CRIBS birth cohort were included in this study (Figure 2). The following maternal characteristics were examined in relation to the food and nutrient intake: age, education, household income, parity, smoking, pre-pregnancy BMI, employment status, working during pregnancy, physical activity level before and during pregnancy, and contraception. Demographic, socioeconomic, and lifestyle data pre- and during pregnancy were collected through two self-completed questionnaires. The medical data were collected from pregnancy booklets and hospital discharge letters, both for mothers and newborns (birth length and weight, delivery mode). Pre-pregnancy BMI was calculated from height and pre-pregnancy weight values, self reported by women, and then compared with measures taken during the first prenatal visit by a trained medical staff. BMI was analyzed as a continuous variable. Education was categorized into two groups: higher education (university and PhD degree) and primary or secondary education (high school or lower levels). Monthly income per family was divided into two categories: ≤10,000 HRK (Croatian currency-kuna) and ≥10,000 HRK, which approximately equals 1300.00 euros of monthly family income. Tobacco consumption was assessed through three questions: (1) whether the mother ever smoked; (2) whether she smoked during pregnancy; (3) and whether she stopped smoking when she found out she was pregnant. Physical activity level at work was divided into low (mostly office work) and high (lifting heavy objects, hard labor) level of work activity. The information about parity was dichotomized into nulliparous or having delivered one or more children previously.

The assessment of dietary intake in pregnancy was determined using the Dietary Adequacy Assessment Questionnaire for Adults (DAAQA), a food frequency questionnaire adapted from the Harvard Semiquantitative Food Frequency Questionnaire. The DAAQA consisted of 101 food items and dietary habits comprising food preparation, food consumption, and dietary supplements habits, together with the frequency of food consumption (usual portion sizes for an individual) [26]. The intakes of different foodstuffs were reported as daily, weekly, or monthly intake frequencies. Adherence to the Mediterranean diet was assessed through two scores—the Mediterranean Diet Serving Score (MDSS) [27] and the Mediterranean Diet Score for pregnant women (MDS-preg [8]. Both scores are simple, valid, and accurate instruments to assess the Mediterranean diet adherence based on the food groups consumption per meal, day, and week. The Mediterranean Diet Serving Score (MDSS) includes 14 food groups, adding 1, 2, or 3 points to the total score based on the consumption frequency and the relative importance of particular type of food, without assigning negative points. According to the proposed MDSS approach, 14 food categories that comprised the Mediterranean diet were created as presented in Table 1.

The maximum possible MDSS score in the original study [27] was 24 points, and the cut-off of ≥13.5 points was considered as good compliance. However, the maximum possible MDSS score in this study was 23 points and the cut-off was set at 12.5 points, since we excluded one category (fermented beverages) from the calculation. An additional advantage of using the MDSS score is the fact that it was already used for the assessment of the Mediterranean diet adherence in southern Croatia in a previous study [18], which enables a comparison between the two datasets. The Mediterranean Diet Score for pregnant women [8], modified from Trichopoulou et al., 2003 (MDS-preg) [28], was also used in evaluating adherence to the Mediterranean diet, since it also does not contain alcoholic beverages (beer or wine), which are usually beneficial in small amounts, but not recommended in pregnancy. We have applied the same thresholds as in previous studies based on the recommendations for pregnant women [28]. For vegetables, fruits, and whole grain products, recommended servings were 3 or more per day, for fish, dairy products, and nuts, the recommended portion was 2 servings or more per day, for legumes over 1.5 servings a week, and for monounsaturated vs. saturated fatty acids ratio recommendation was to exide 1.6. Those recommendations were assigned the value of 1, otherwise they were assigned 0 points. The consumption of red and processed meat under the threshold (lower than 4.5 servings weekly) was assigned the value of 1, and higher amounts 0 points. The MDS ranged from 0–3 represents low adherence, a score of 4–6 represents moderate adherence, and a score of 7–9 represents high adherence to the Mediterranean diet.

All data preprocessing procedures were written in Python 3.7, Python Software Foundation, Delaware, United States. Raw data from polls were converted to numerical values by calculating frequencies of the individual variables related to their daily frequencies, e.g., two times per week will be converted to numerical value 2/7. We removed the variables with constant values, high inter-correlation, missing values, or very low variance. The calculated MDSS scores were joined with the data. We calculated cumulative frequencies from the nutrition questionnaire by summing the frequencies across participants (row-wise) for similar answers, e.g., the frequency of eating white meat or the frequency of eating vegetables. The cumulative frequencies were used in further analysis. The final data set consists of 266 participants and 105 variables.

The non-parametric Mann–Whitney U (MWU) test was utilized for statistical comparisons between the two target groups divided by location (islands/mainland). The hypotheses are defined as follows: H0: “There is no difference in the dietary habits between island and mainland participants”; H1: “There are differences in the dietary habits between island and mainland participants” across nutritional habits and lifestyle. The two dataset groups were compared with all the variables from preprocessed data. This increased the risk of obtaining positive results on the basis of chance alone. To counteract this risk, we employed the Bonferroni correction for multiple testing. The Bonferroni correction [29] involves choosing an overall alpha and dividing this by the number of tests to be conducted. This results in a corrected level of significance for each tested variable. Furthermore, we used principal component analysis (PCA) [30] to reveal potential nutritional patterns in food groups/summarized food frequencies [31]. For that purpose, we scaled the cumulative frequency data and plotted the PCA loadings.

To understand potential non-linear and complex relationships besides statistical difference, we employed machine learning (ML), i.e., random forest (RF) classification [32]. An ensemble classifier, i.e., random forest had showed good performance before in analyzing nutritional patterns [33]. Besides their excellent performance, tree-based machine learning methods do not need heavy data preprocessing and are convenient for the work with heterogeneous and imbalanced data [34]. Our concept was to train a classifier trying to resolve a two-class problem, namely the assignment of whether a participant lives on an island or in the mainland region (class 0 = island or class 1 = mainland). RF has an internal variable importance option which can reveal information on important predictors such as lifestyle or nutrition in the trained classifier [33]. The variable importance in RF is an averaged value of the most important variables when splitting a decision tree inside the random forest. The predictive variables for the classifier are the aforementioned data from the questionnaire. The models are validated by means of calculating the misclassification error on unobserved participants. The data is therefore randomly split on a train (75%) and test set (25%). The models are trained on the train set and validated on the test set with several metrics 1000 times, ensuring a different random split every time. A model quality metric used often in classification, which is accuracy, is ineffective in cases with a minor and major class [35]. The following metrics are commonly accepted in imbalanced classification: sensitivity, specificity, auc, the Kappa Cohen score [36], and the Matthews correlation coefficient [37,38]. Sensitivity is a measure of completeness (i.e., number of correctly classified true positives, here class 1), while specificity represents the true negative rate (class 0 correctly classified as class 0). The Matthews correlation coefficient is a correlation coefficient for binary data and sensitive to the misclassification in imbalanced data. Except MCC, which ranges in values −1, 1 with 1 being a perfect classifier, the other metrics range in values 0.1.

## 3. Results

### 3.1. Results of the Statistical Analysis

The mean age of CRIBS participants in this study was 30.1 years of age. Their baseline characteristics with regards to the place of residence (island vs. mainland) are shown in Table 2. Most of the significant differences (*p* < 0.001) between women from the mainland and from the islands have been observed in the context of education, income, physical activity before pregnancy, and work activity. Namely, women from the islands have lower education and household budget, but higher activity levels. If they are employed, their activity level at work is higher than in pregnant women from the mainland and they report higher activity before pregnancy in general. Their activity mostly involves agricultural work and intensive housework activities in the tourism sector. On the other hand, women from the mainland work more often in the business and educational sector and have other sedentary occupations where physical activity is known to be reduced. Additionally, women from the islands have more children (higher parity) (*p* < 0.01), a much lower contraception rate (*p* < 0.01), and smoke more often than women from the mainland (*p* < 0.05). A trend of higher pre-pregnancy BMI values has been observed on the islands and more than 25% of islanders are in the overweight/obese category, which raises concern.

Table 3 shows mainland–island differences in maternal lifestyle, namely pre-pregnancy BMI, parity, contraception level, and smoking, with regards to the variables that most significantly differentiate CRIBS women according to the location (Table 2)—education level, monthly income, pre-pregnancy activity, and work activity. The results indicate that higher parity is correlated to lower educational attainment (*p* < 0.001) and smaller household budget (*p* < 0.01), and islanders in general report less contraception use (*p* < 0.01). No significant difference between pre-pregnancy BMI and education has been detected, but the islanders with a higher income have a higher BMI before pregnancy (*p* < 0.01). With regards to pre-pregnancy activity and work activity, pregnant women from the islands that are more active still have higher pre-pregnancy BMI values than the same sample from the mainland (*p* < 0.01). The prevalence of smoking in pregnancy is significantly higher on the islands, especially among wealthier women (*p* < 0.05). In addition, women from the islands that report a low level of physical activity at work have significantly higher pre-pregnancy BMI values and smoke more often (*p* < 0.01).

Only 27.8% of the study participants adhere to the Mediterranean dietary pattern according to MDSS results. The median MDSS score was 10.35 out of 23 points. By means of the MDS-preg score, we observe a higher adherence - 51.5% of CRIBS pregnant women adhere moderately or highly to the Mediterranean dietary pattern. The median MDS-preg score was 3.63 out of 10 points (Appendix A Table A1 and Table A2). The participants reported a dietary pattern that had especially low compliance with the Mediterranean diet guidelines for consumption of sweets (only 3% met the criteria), red meat (only 8% met the criteria), fish (only 22% met the criteria), nuts (19% met the criteria), and moderate consumption of vegetables (47% met the criteria), based on MDSS score. Based on MDS-preg score, low compliance was recorded for red meat (22% met the criteria), fish (25% met the criteria), and vegetables (19% met the criteria). Altogether, the participants in the CRIBS study eat too much red meat and sweets and too small amounts of fish and vegetables (Appendix A Table A1 and Table A2).

Only marginal differences have been observed regarding MDSS scores between women from the islands and those from the mainland. Even though there is a slight geographical difference, they belong to the same sociocultural group with similar dietary habits. However, it is obvious that women with higher educational levels and lower BMI on both islands and mainland adhere more to the Mediterranean dietary pattern according to both scores, as presented in Table 4. Significant island–mainland difference has been observed only in the MDSS score regarding the household budget. Namely, wealthier women on both islands and the mainland adhere more to the Mediterranean diet (*p* < 0.05). A connection between Mediterranean diet scores and delivery mode, complications in pregnancy, or child anthropometry has not been detected.

**Figure 3:**
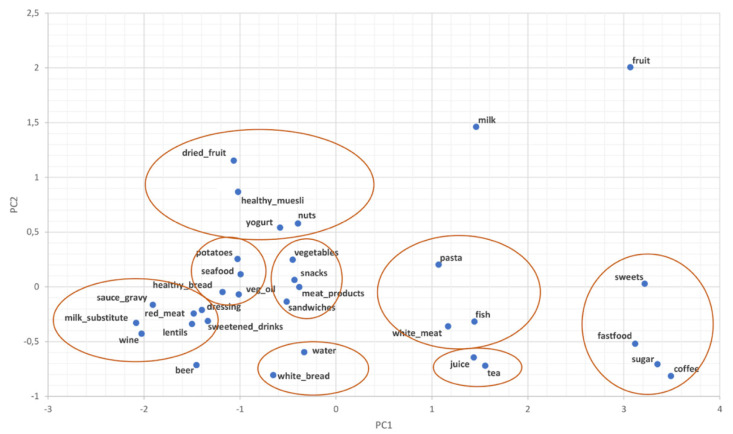
Principal component analysis (PCA) loadings plot of the cumulative frequencies of food intake.

To reveal nutritional patterns in more detail, we conducted a principal component analysis (PCA) on cumulative frequencies of food items obtained from the nutritional questionnaire. The patterns are shown by means of the PCA loadings plot (Figure 3). PC1 appears to represent an abstraction of sugar and fat load in the sample with juice, alcoholic beverages, snacks, and meat giving a higher positive load on PC1, the rather balanced food without low loads on PC1, and fatty/heavier food showing a high negative load on PC1. PC2 appears to mirror clustering of food items in concordance to the main daily meals, with typical breakfast (muesli, yogurt, nuts, dried fruit) having a high load on PC2, heavy lunch food items (read meat, sauce, wine, potatoes, white bread) with almost no load on PC2, and basic food items (water, white bread), drinks, and coffee as light meal options with a negative load on PC2. The results indicate that women who eat more sweets, also eat more fast food and drink coffee. Similarly, women who eat red meat frequently also consume more sauces, gravies, and dressings and drink more wine and sweetened drinks—all food items that can be classified as unhealthy and not recommended during pregnancy. Two healthy groups are evident as well, encompassing typical food items of the Mediterranean diet (pasta, fish, and white meat in one group and dried fruit, muesli, yogurt, and nuts in the other group).

Table 5 shows mainland–island differences in the consumption of different food groups, with regards to the variables that most significantly differentiate CRIBS women according to the location (Table 2)—education level, monthly income, pre-pregnancy activity, and work activity. Our results show statistically significant values in consumption of beans and lentils in a group of women with high pre-pregnancy BMI (*p* < 0.001), where islanders consume it somewhat more in their diet. In addition, it seems that the same group (high BMI) of women eats more fast food (*p* < 0.01), which is also connected to low daily sitting pre-pregnancy (*p* < 0.001) and low work activities in both mainlanders and islanders (*p* < 0.01). On the other hand, women from the mainland report higher consumption of fish, nuts, and fruits, which is connected to higher monthly household budget (*p* < 0.01 and *p* < 0.05 respectively), confirming that adherence to the Mediterannean diet is probably connected to socio-economic status, rather than geographical location, and mostly consumed among wealthier women. In addition, mainlanders with low work activity, but high general pre-pregnancy activity seem to consume more healthy muesli (*p* < 0.01). The consumption of healthy bread is connected to low pre-pregnancy BMI (*p* < 0.001), high pre-pregnancy activity (*p* < 0.001), and work activity (*p* < 0.01) in both mainlanders and islanders. Additionally, more educated women eat less bread in general (*p* < 0.05 and *p* < 0.01, respectively), while the opposite trend has been observed for the ones with a low educational level (*p* < 0.05) and household budget (*p* < 0.01).

### 3.2. Classification Results

**Figure 4:**
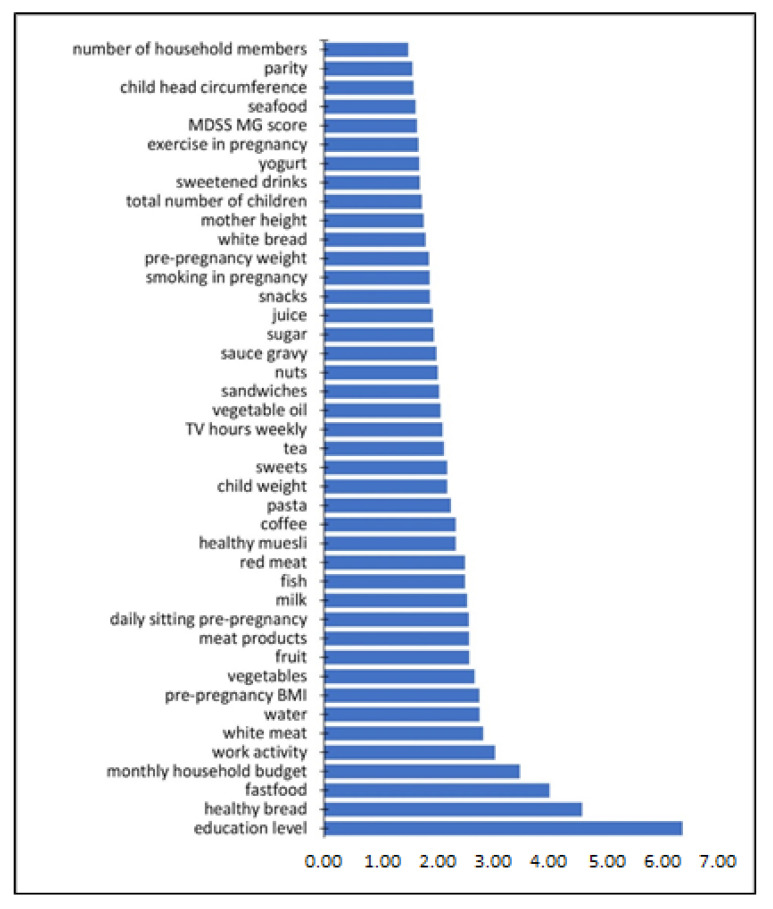
Variable importance in the random forest classifier.

In order to extract variables which separate the two classes (island vs. mainland) in a non-linear manner, a machine learning approach was used. The results from the 1000 trained RF classifiers are reported as mean with a 95% confidence interval. With all of the variables (104) employed, the following classification metrics were obtained: accuracy 0.69 ± 0.01, kappa cohen score 0.35 ± 0.02; mcc 0.36 ± 0.02; auc score 0.74 ± 0.01; sensitivity 0.58 ± 0.02; specificity 0.78 ± 0.02. Judging by the values of kappa cohen score and the mcc, the classification can be categorized as “fair” [35]. The full variable importance table can be found in Appendix A Table A3. Variable importance was used as a tool for variable selection [39]. Variables with a mean importance of 1% and above were selected as relevant. There were 42 selected variables (Appendix A Table A4) which were employed in re-training. The re-training with re-sampling of 1000 classifiers after variable selection resulted in an insignificant improvement of the classification metrics. The results were: accuracy 0.69 ± 0.01, kappa cohen score 0.37 ± 0.02, mcc 0.38 ± 0.02, auc score 0.75 ± 0.01, sensitivity 0.59 ± 0.02, and specificity 0.77 ± 0.02. The 42 variable importances are plotted in a bar plot in Figure 4. Some dominant variables are the education level, followed by the cumulative frequencies in eating (healthy) bread and fast food, household budget, work activity, cumulative consumption of white meat, and hydration, as well as pre-pregnancy BMI.

## 4. Discussion

This study was designed to identify the level of adherence to the Mediterranean diet and recommended lifestyle habits within a cohort of pregnant women from the Dalmatian region of Croatia (the CRIBS cohort). Our study suggests that the current globalization trends have influenced island communities in the same way as the mainland and that they form a rather homogenous group with regards to dietary patterns, which are characterized by low to moderate adherence to the Mediterranean diet. However, significant differences between the two groups of participants (mainland vs. island) were highlighted for lifestyle and socioeconomic factors. The largest differences were evident in education and income level, pre-pregnancy sedentary behavior, work activity, parity, as well as the usage of contraception. Less significant differences were noticed for smoking habits and pre-pregnancy BMI. When divided according to location and compared to socioeconomic factors, only marginal differences regarding the Mediterranean diet scores were reported in our study. Interestingly, the highest adherence to the Mediterranean diet was observed among wealthier women on both islands and mainland, highlighting that healthy behavior could be more connected to socio-economic status than to the pregnancy itself. Evidence from previous studies also suggested that educational level and income were positively associated with the adherence to the Mediterranean diet or to a healthy dietary pattern during pregnancy in general [11,40,41].

A conventional statistical approach (MWU test) in this study was integrated with supervised machine learning (ML), the results of which were mostly in agreement with the standard approach. Even though there is only a marginal improvement in the model metrics due to the variable selection, the spaces of these two groups can be separated with as few as 42 variables, but a perfect separation is not possible. This again indicates that a mainland–island differentiation is present, but that these groups still belong to the same geographical area, with a population exchange that allows its overall homogeneity. The ML results show a higher specificity than sensitivity, meaning there is less misclassification for the mainland and possibly a larger homogeneity within this group. Furthermore, there is a concordance between both methods on the relevant variables such as education level, household budget, and working activity. Machine learning also highlighted certain nutritional variables as important for mainland–island differentiation, which were later also confirmed in additional statistical tests (cumulative frequencies of healthy bread and healthy muesli consumption, fast food and white meat consumption). We wanted to stress the importance of using both statistical tests and non-linear machine learning methods when analyzing such problems. Even though statistical tests are indispensable when comparing groups, machine learning can reveal additional discrimination between groups hidden in the data, that might reflect subgroups in a multivariate space.

Croatian pregnant women in this study demonstrated low to moderate compliance with the Mediterranean diet, although healthy dietary habits should be a high priority during pregnancy according to current recommendations [12]. They consume more red meat and sweets and less fish and vegetables, as expected according to both general Mediterranean diet score (MDSS) and the adjusted one for pregnant women (MDS-preg). This corresponds to an unsatisfactory Mediterranean diet consumption level in southern Croatia which was suggested already on a sample from the general population by Kolčić et al. (2016) and with the overall decline in adherence to Mediterranean eating patterns in other Mediterranean island population [42]. A more detailed insight into the relationship between individual food items and their cumulative frequencies identified several clusters by employing an unsupervised machine learning method i.e., PCA. It showed that healthy and unhealthy food tends to cluster together, forming traditional, healthy groups and groups that can be characterized as “Westernized”, modern, and unhealthy. Although our study group includes solely pregnant women, unhealthy dietary patterns and consumption of heavy food items were quite pronounced. This again suggests that, although women are aware of the importance of nutrition during pregnancy, they often do not improve their dietary habits. In addition, this implies that globalization does not reflect itself only in the introduction of certain items but rather in the change of the whole lifestyle. Similar results on poor compliance with healthy types of nutrition and drifting away from Mediterranean dietary habits have also been reported among pregnant women in other Mediterranean countries [13,43].

A detailed analysis of the mainland–island differences in the consumption of specific food items revealed that mainland women, especially more educated and wealthier ones, have healthier eating habits (e.g., consummation of more fish, nuts and fruits, and less bread). Even though it would be expected that islanders would follow the traditional Mediterranean lifestyle and diet due to easier access to the healthy components of the Mediterranean diet (such as fish, wine, and olive oil), a significant increase in the consumption of red meat, poultry, milk, dairy products, sugar, and industrial products has been observed in this subset of pregnant women, as well as in previous studies conducted in this region [17,18,20]. One of the key features of the globalization phenomenon, namely the neglect of agriculture and the appearance of supermarkets on the islands, is directing people towards the consumption of cheaper, more easily accessible, high-energy, and nutrient-poor food. Such diet consequently has considerable effect on the health of the population (e.g., higher BMI values), which we have confirmed in this study. Interestingly, islanders with a higher income also have higher BMI values than women from urban areas, although they are generally more active. This could potentially be explained by a theory proposed by Missoni and collaborators that pasta, fish, and black bread have been perceived as symbols of poverty, while red meat and white bread were considered elite food. Nowadays, when these so-called elite-products are equally available, people from Croatian insular, rural regions turn to them more often [17]. Even though the adherence to the Mediterranean diet was reported to be beneficial for the newborn body size or delivery mode in some other studies [9], it did not show significant correlation in our study.

The differences in lifestyle between the two subsets of pregnant women (islands vs. mainland) were also confirmed with regards to reproduction, physical activity, and smoking. More than 20% of CRIBS participants in general and more than 30% of women from the islands continued smoking during pregnancy, especially wealthier ones, even though it is a known risk factor for adverse pregnancy outcomes and early child growth and development. Identified smoking patterns among pregnant women within this study mirrored smoking behavior in the general Croatian population of women and were only slightly lower [44,45]. Furthermore, the observed lower rates of contraception and higher parity on the islands indicate low levels of family planning and poor access to reproductive health services, which is more common in traditional communities [22]. In general, more women from the mainland are employed when compared to the island participants and the differences are related also to their working activities. Higher overall workload reported for women from the islands is expected as schools and office jobs are less available and the economy is rather based on agriculture and tourism. Islanders are also more physically active in general, which could potentially be observed as a protective factor for their general health, despite certain negative lifestyle trends (such as smoking) and increased pre-pregnancy BMI values. Other European studies also reported similar associations between smoking, unhealthy dietary patterns, and high BMI values among pregnant women [11].

In conclusion, analysis of data in our study highlighted inappropriate nutritional behavior and lifestyle habits among pregnant women in the Mediterranean part of Croatia, which is not in accordance with the Croatian nutritional guidelines for adults or global dietary recommendations during pregnancy [12,16]. The highest adherence to the Mediterranean diet was observed among wealthier women with generally healthier lifestyle choices in both locations. Adverse socioeconomic and lifestyle conditions, leading to the adoption of behaviors which contribute to poor health (such as poor diet or non-Mediterranean dietary pattern) were especially observed among pregnant women in island populations. This fact strongly confirms that the insular part of Croatia is represented by communities with a greater burden of CVD risk factors, higher BMI values, and overall low adherence to the Mediterranean diet, as reported in previous studies [19,24,46,47]. Identifying the factors associated with changes in dietary patterns and lifestyle in pregnant women may help focus on more vulnerable populations and encourage them against poor eating choices and lifestyle habits. Public health strategies (giving priority to nutrition policy objectives, development of specific pregnancy-related guidelines, educational workshops) aiming to improve adherence to the Mediterranean dietary pattern and to promote healthy behavior should be promoted more intensely, especially in smaller rural and island communities in Croatia.

The major strength of our study lies in the fact that it presents the first data on adherence to the Mediterranean diet in pregnant islanders from Dalmatia, Croatia. The comparison with neighboring mainland counterpart is also demonstrated. However, there are some limitations to our study. The major limitation of the study is a relatively small number of participants. However, when the general population size of Croatia is taken into consideration, together with the very low pregnancy rate on the Adriatic islands, our sample of 266 participants (half of them from the islands), could be considered sufficient. Second, the use of FFQ and self-reported lifestyle behaviors could introduce possible bias, especially with regards to undesirable lifestyle behaviors in pregnancy, such as smoking or alcohol intake. Further research including randomized control trials on larger samples from Mediterranean and continental regions of Croatia are needed to identify other possible predictors associated with maternal health during pregnancy and the adherence to Mediterranean diet in particular.

## Figures and Tables

**Table 1:** Food categories used for the calculation of the Mediterranean Diet Serving Score (MDSS).

Category	Serving	Score	Included Food Items
fresh fruit	1–2 servings/main meal	3	fresh fruit
vegetables	≥2 servings/main meal	3	vegetables in main dish, vegetables in side dish, fresh vegetables, frozen vegetables, vegetables in stew/soup/wok
cereals	1–2 servings/main meal	3	healthy bread (wheat bread, black bread, whole wheat bread, rye bread, corn bread, multigrain bread), unhealthy bread (white bread, toast), rice, pasta, healthy muesli (sugar-free/unsweetened muesli, oatmeal, cornflakes), unhealthy muesli (sweetened muesli, choco balls)
potatoes	≤3 servings/week	1	mashed potatoes, baked potatoes, french fries
olive oil	1 serving/main meal	3	olive oil
nuts	1–2 servings/day	2	nuts, walnuts, almonds
dairy products	2 servings/week	2	high-fat milk, average-fat milk, low-fat milk, high-fat yogurt, low-fat yogurt, fruit yogurt, kefir
legumes	≥2 servings/week	1	legumes
eggs	2–4 servings/week	1	eggs
fish	≥2 servings/week	1	blue fish, white fish, shellfish, squid/octopus, crabs
white meat	2 servings/week	1	poultry, turkey
red meat	<2 servings/week	1	beef/veal, pork, lamb, venison, sausages, hotdogs
sweets	≤2 servings/week	1	pudding, canned fruit, ice cream, sweet pastries, donuts, waffles, chocolate cake, cookies, pancakes, chocolate, candies, jelly, marcipan, fruit syrup, coca cola, other carbonated soft drinks, other soft sweetened drinks, energy drinks, vitamin drinks
fermented beverages	1–2 glass/day	0 *	wine, beer, spirits

* Excluded from the calculation, since the tested population were pregnant women that reported no alcohol consumption due to their pregnancy.

**Table 2:** Baseline characteristics of Croatian Islands’ Birth Cohort Study (CRIBS) participants by place of residence (* *p* = 0.056, ** *p* < 0.05, *** *p* < 0.01, **** *p* < 0.001). The results are obtained by means of the Mann–Whitney U (MWU) test.

Maternal Characteristics		Location	
		Island *N* (%)	Mainland *N* (%)
Education ****	high	46 (30.9)	78 (66.7)
	low	103 (69.1)	39 (33.3)
Income ****	high	25 (16.8)	52 (44.4)
	low	124 (83.2)	65 (55.6)
Pre-pregnancy sitting daily ****	high	3 (2.0)	11 (9.4)
	low	146 (98.0)	106 (90.6)
Work activity ****	high	53 (35.6)	20 (17.1)
	low	96 (64.4)	97 (82.9)
Parity ***	primipara	46 (30.9)	60 (51.3)
	multipara	103 (69.1)	57 (48.7)
Contraception ***	yes	18 (12.5)	32 (27.4)
	no	126 (87.5)	85 (72.6)
Smoking **	yes	45 (30.2)	17 (14.5)
	ex smoker	45 (30.2)	48 (41.0)
	never	59 (39.6)	52 (44.5)
Pre-pregnancy BMI *	underweight	19 (12.9)	13 (11.2)
	normal	90 (61.2)	86 (74.1)
	overweight/obese	38 (25.9)	17 (14.7)

**Table 3:** Differences in maternal socioeconomic factors and lifestyle with regards to location (mean values are converted to high-low values with level of significance for the location tested by the Mann–Whitney U (MWU) test; * *p* < 0.05, ** *p* < 0.01, *** *p* < 0.001; only cumulative frequencies with at least one significant difference were taken into consideration; ′ the test has not been performed for this group, since the number of individuals was <5).

Location	2nd Var	N of Participants	Value	Children Total	Contra-Ception	Pre-PregBMI	Smoking
Mainland	education level ***	78	High	0.50 ***	0.36	20.45	0.53
Island		46	High	0.93 ***	0.24	21.46	1.15
Mainland		39	Low	0.74	0.36 **	21.50	1.21
Island		103	Low	0.99	0.17 **	22.92	2.97
Mainland	household budget monthly ***	52	High	0.58 **	0.38	20.42 **	0.56 *
Island		25	High	1.04 **	0.28	23.09 **	1.64 *
Mainland		65	Low	0.58 **	0.34 **	21.10	0.91
Island		124	Low	0.96 **	0.17 **	22.34	2.56
Mainland	sitting pre-pregnancy daily ***	11	High	0.09	0.27	22.71	0.45
Island		3′	High	0.67	0.33	25.91	0
Mainland		106	Low	0.63 ***	0.37 ***	20.60 **	0.78 **
Island		146	Low	0.98 ***	0.18 ***	22.39 **	2.46 **
Mainland	work activity ***	20	High	0.60	0.30	22.26	1.45
Island		53	High	0.94	0.19	21.91	2.53
Mainland		97	Low	0.58 ***	0.37 **	20.50 **	0.61 **
Island		96	Low	0.99 ***	0.19 **	22.77 **	2.34 **

**Table 4:** Adherence to the Mediterranean diet according to two Mediterranean diet scores (* *p* < 0.05).

Location	2nd Variable	Value	MDSS Score	MDS-Preg Score	N of Participants
Mainland	Education level	High	10.97	3.78	78
Island		High	11.33	3.93	46
Mainland		Low	10.1	3.44	39
Island		Low	9.53	3.46	103
Mainland	Monthly household budget	High	11.48 *	4.04	52
Island		High	10.16 *	3.76	25
Mainland		Low	10.05	3.37	65
Island		Low	10.07	3.57	124
Mainland	Pre-pregnancy BMI	High	9.65	3.06	17
Island		High	9.74	3.29	38
Mainland		Low	10.86	3.77	100
Island		Low	10.21	3.71	111

**Table 5:** Cumulative frequencies of food items with regards to location and other relevant maternal socioeconomic and lifestyle factors (mean values are converted to high-low values with level of significance for the location tested by the Mann–Whitney U (MWU) test (* *p* < 0.05, ** *p* < 0.01, *** *p* < 0.001; only cumulative frequencies with at least one significant difference were taken into consideration).

Location	Variable	Value	*N* of Participants	Nuts	Snacks	Fruit	Dressing	Beans Lentils	Fast Food	Vegetables	Fish	White Meat	Meat Products	Tea	Sweet. Drinks	Sugar	Unhealthy Muesli	Healthy Muesli	Healthy Bread
Mainland	education level	High	78	0.34	0.16	7.42	0.16 *	0.06	1.07	5.28	2.85	0.35	0.31	3.83	0.3	7.14	0.07	0.29 *	0.41 *
Island		High	46	0.19	0.12	6.8	0.25 *	0.06	1.04	4.97	2.73	0.3	0.27	3.88	0.29	6.4	0.05	0.19 *	0.43 *
Mainland		Low	39	0.23	0.16	7.9	0.12	0.04 *	1.17	4.7	2.64	0.32	0.43	3.95	0.6	7.33	0.16	0.37	0.71 *
Island		Low	103	0.25	0.13	6.86	0.2	0.05 *	1.17	5.1	2.62	0.28	0.35	4.22	0.47	7.64	0.08	0.23	0.64 *
Mainland	monthly household budget	High	52	0.36 *	0.13	7.95 *	0.11 **	0.07	1.09 *	5.19	2.97 **	0.31	0.34	3.54 *	0.37	6.8 *	0.11	0.34	0.46
Island		High	25	0.18 *	0.11	6.39 *	0.23 **	0.05	1.04 *	5.42	2.57 **	0.25	0.26	4.24 *	0.63	7.74 *	0.02	0.09	0.47
Mainland		Low	65	0.26	0.18	7.29	0.17	0.05	1.11 *	5	2.63	0.36	0.35	4.13	0.43	7.53	0.09	0.29 *	0.55 **
Island		Low	124	0.24	0.13	6.94	0.21	0.06	1.15 *	4.99	2.67	0.29	0.34	4.09	0.37	7.16	0.08	0.24 *	0.6 **
Mainland	pre-pregnancy BMI	High	17	0.21	0.11	6.18	0.11	0.02 ***	1.11 **	4.49	3	0.31	0.42	3.71	0.44	7.14	0.06	0.18	0.47
Island		High	38	0.17	0.12	6.03	0.29	0.07 ***	1.25 **	5.54	2.76	0.26	0.38	3.9	0.42	8.01	0.09	0.14	0.79
Mainland		Low	100	0.32	0.16	7.82	0.15	0.06	1.1 *	5.19	2.74	0.34	0.33	3.9	0.39	7.22	0.11	0.34 *	0.52 ***
Island		Low	111	0.25	0.13	7.12	0.19	0.05	1.09 *	4.9	2.62	0.3	0.31	4.19	0.42	7	0.07	0.25 *	0.5 ***
Mainland	daily sitting pre-pregnancy	High	11	0.36	0.12	7.93	0.12	0.03	1.21	4.32	2.78	0.46 *	0.43	3.6	0.14	6.72	0.11	0.42	0.41
Island		High	3	0.15	0.1	5.86	0.06	0.02	1.11	4.46	2.21	0.17 *	0.14	3.11	0.22	5.39	0.01	0.03	0.23
Mainland		Low	106	0.3 *	0.16	7.54	0.15	0.06	1.09 ***	5.16	2.78 *	0.33	0.34 *	3.9	0.43	7.26	0.1 *	0.3 **	0.52 ***
Island		Low	146	0.23 *	0.13	6.87	0.22	0.06	1.13 ***	5.07	2.66 *	0.29	0.33 *	4.14	0.42	7.29	0.07 *	0.22 **	0.58 ***
Mainland	work activity	High	20	0.25	0.21 *	7.54	0.11	0.03 *	1.1	4.51	2.77	0.28	0.46	4.07	0.27	7.71	0.09	0.3	0.47 *
Island		High	53	0.28	0.12 *	6.76	0.31	0.05 *	1.16	5.61	2.7	0.28	0.38	3.88	0.27	7.27	0.06	0.27	0.63 *
Mainland		Low	97	0.32 *	0.15	7.59	0.15	0.06	1.1 **	5.2 *	2.79 *	0.35 *	0.32	3.83 *	0.43 *	7.1	0.1	0.32 **	0.52 **
Island		Low	96	0.2 *	0.13	6.89	0.16	0.06	1.11 **	4.76 *	2.63 *	0.29 *	0.29	4.25 *	0.49 *	7.25	0.08	0.19 **	0.54 **

## Appendix A

**Table A1:** MDSS score calculation according to specific food categories.

ID	Location	Cereal	Eggs	Dairy	White_Meat	Red_Meat	Fish	Legumes	Potatoes	Olive	Vegetables	Fruit	Nuts	Sweets	Score	Category (Cut-Off 12.5)	Adherence
ID266	2.00	3.00	0.00	0.00	0.00	0.00	0.00	0.00	0.00	0.00	0.00	0.00	0.00	0.00	3.00	1.00	low
ID49	2.00	3.00	1.00	0.00	0.00	0.00	0.00	0.00	0.00	0.00	0.00	0.00	0.00	0.00	4.00	1.00	low
ID93	2.00	3.00	0.00	0.00	0.00	0.00	0.00	0.00	0.00	0.00	0.00	0.00	0.00	1.00	4.00	1.00	low
ID133	2.00	3.00	0.00	0.00	0.00	0.00	0.00	0.00	1.00	0.00	0.00	0.00	0.00	0.00	4.00	1.00	low
ID242	2.00	3.00	1.00	0.00	0.00	0.00	0.00	0.00	0.00	0.00	0.00	0.00	0.00	0.00	4.00	1.00	low
ID249	2.00	3.00	0.00	0.00	1.00	0.00	0.00	0.00	0.00	0.00	0.00	0.00	0.00	0.00	4.00	1.00	low
ID257	2.00	3.00	1.00	0.00	0.00	0.00	0.00	0.00	0.00	0.00	0.00	0.00	0.00	0.00	4.00	1.00	low
ID94	2.00	3.00	0.00	0.00	0.00	0.00	0.00	0.00	0.00	0.00	0.00	0.00	2.00	0.00	5.00	1.00	low
ID97	2.00	0.00	1.00	0.00	1.00	0.00	0.00	0.00	0.00	0.00	0.00	3.00	0.00	0.00	5.00	1.00	low
ID118	2.00	3.00	1.00	0.00	1.00	0.00	0.00	0.00	0.00	0.00	0.00	0.00	0.00	0.00	5.00	1.00	low
ID137	1.00	3.00	1.00	0.00	1.00	0.00	0.00	0.00	0.00	0.00	0.00	0.00	0.00	0.00	5.00	1.00	low
ID142	1.00	3.00	1.00	0.00	0.00	0.00	0.00	1.00	0.00	0.00	0.00	0.00	0.00	0.00	5.00	1.00	low
ID194	1.00	3.00	0.00	2.00	0.00	0.00	0.00	0.00	0.00	0.00	0.00	0.00	0.00	0.00	5.00	1.00	low
ID199	1.00	3.00	1.00	0.00	0.00	0.00	1.00	0.00	0.00	0.00	0.00	0.00	0.00	0.00	5.00	1.00	low
ID251	2.00	3.00	1.00	0.00	0.00	0.00	0.00	0.00	1.00	0.00	0.00	0.00	0.00	0.00	5.00	1.00	low
ID5	1.00	3.00	0.00	0.00	0.00	0.00	0.00	0.00	0.00	0.00	0.00	3.00	0.00	0.00	6.00	1.00	low
ID74	2.00	3.00	1.00	0.00	0.00	0.00	1.00	0.00	1.00	0.00	0.00	0.00	0.00	0.00	6.00	1.00	low
ID103	2.00	3.00	0.00	0.00	0.00	0.00	0.00	0.00	0.00	0.00	0.00	3.00	0.00	0.00	6.00	1.00	low
ID106	2.00	0.00	0.00	0.00	0.00	1.00	1.00	0.00	1.00	0.00	0.00	3.00	0.00	0.00	6.00	1.00	low
ID123	2.00	3.00	0.00	0.00	0.00	0.00	0.00	0.00	0.00	0.00	0.00	3.00	0.00	0.00	6.00	1.00	low
ID125	2.00	3.00	0.00	0.00	0.00	0.00	0.00	0.00	0.00	0.00	0.00	3.00	0.00	0.00	6.00	1.00	low
ID145	1.00	3.00	1.00	0.00	0.00	0.00	0.00	1.00	1.00	0.00	0.00	0.00	0.00	0.00	6.00	1.00	low
ID156	1.00	3.00	1.00	0.00	1.00	0.00	1.00	0.00	0.00	0.00	0.00	0.00	0.00	0.00	6.00	1.00	low
ID163	1.00	3.00	0.00	0.00	0.00	0.00	0.00	0.00	0.00	0.00	0.00	3.00	0.00	0.00	6.00	1.00	low
ID196	1.00	3.00	0.00	0.00	0.00	0.00	0.00	0.00	0.00	0.00	0.00	3.00	0.00	0.00	6.00	1.00	low
ID210	1.00	3.00	0.00	0.00	0.00	0.00	0.00	0.00	0.00	0.00	0.00	3.00	0.00	0.00	6.00	1.00	low
ID3	1.00	3.00	0.00	0.00	0.00	0.00	0.00	0.00	1.00	0.00	0.00	3.00	0.00	0.00	7.00	1.00	low
ID14	1.00	3.00	1.00	2.00	0.00	0.00	0.00	0.00	1.00	0.00	0.00	0.00	0.00	0.00	7.00	1.00	low
ID18	1.00	0.00	1.00	0.00	1.00	1.00	0.00	0.00	1.00	0.00	0.00	3.00	0.00	0.00	7.00	1.00	low
ID30	2.00	3.00	1.00	0.00	0.00	0.00	0.00	0.00	0.00	0.00	0.00	3.00	0.00	0.00	7.00	1.00	low
ID46	2.00	3.00	1.00	0.00	0.00	0.00	0.00	0.00	0.00	0.00	0.00	3.00	0.00	0.00	7.00	1.00	low
ID47	2.00	3.00	1.00	0.00	0.00	0.00	0.00	0.00	0.00	0.00	0.00	3.00	0.00	0.00	7.00	1.00	low
ID50	2.00	3.00	1.00	0.00	0.00	0.00	0.00	0.00	0.00	0.00	0.00	3.00	0.00	0.00	7.00	1.00	low
ID55	2.00	3.00	1.00	0.00	0.00	0.00	0.00	0.00	0.00	0.00	0.00	3.00	0.00	0.00	7.00	1.00	low
ID60	2.00	3.00	1.00	2.00	0.00	0.00	1.00	0.00	0.00	0.00	0.00	0.00	0.00	0.00	7.00	1.00	low
ID64	2.00	3.00	1.00	0.00	0.00	0.00	0.00	0.00	0.00	0.00	0.00	3.00	0.00	0.00	7.00	1.00	low
ID70	2.00	3.00	0.00	0.00	0.00	0.00	0.00	0.00	1.00	0.00	0.00	3.00	0.00	0.00	7.00	1.00	low
ID71	2.00	3.00	1.00	0.00	0.00	0.00	0.00	0.00	0.00	0.00	0.00	3.00	0.00	0.00	7.00	1.00	low
ID80	2.00	3.00	0.00	0.00	0.00	0.00	0.00	0.00	1.00	0.00	0.00	3.00	0.00	0.00	7.00	1.00	low
ID95	2.00	3.00	1.00	0.00	0.00	0.00	0.00	0.00	0.00	0.00	0.00	3.00	0.00	0.00	7.00	1.00	low
ID100	2.00	3.00	1.00	0.00	0.00	0.00	0.00	0.00	0.00	0.00	0.00	3.00	0.00	0.00	7.00	1.00	low
ID102	2.00	3.00	1.00	0.00	0.00	0.00	0.00	0.00	0.00	0.00	0.00	3.00	0.00	0.00	7.00	1.00	low
ID114	2.00	3.00	0.00	0.00	0.00	0.00	0.00	0.00	1.00	0.00	0.00	3.00	0.00	0.00	7.00	1.00	low
ID120	2.00	3.00	1.00	0.00	0.00	0.00	0.00	0.00	0.00	0.00	0.00	3.00	0.00	0.00	7.00	1.00	low
ID126	2.00	3.00	0.00	0.00	1.00	0.00	0.00	0.00	0.00	0.00	0.00	3.00	0.00	0.00	7.00	1.00	low
ID127	2.00	0.00	0.00	0.00	0.00	0.00	1.00	0.00	0.00	0.00	3.00	3.00	0.00	0.00	7.00	1.00	low
ID168	1.00	3.00	1.00	2.00	0.00	0.00	0.00	0.00	1.00	0.00	0.00	0.00	0.00	0.00	7.00	1.00	low
ID178	1.00	3.00	1.00	0.00	0.00	0.00	0.00	0.00	0.00	0.00	0.00	3.00	0.00	0.00	7.00	1.00	low
ID186	1.00	3.00	0.00	0.00	0.00	0.00	0.00	0.00	1.00	0.00	0.00	3.00	0.00	0.00	7.00	1.00	low
ID195	1.00	3.00	1.00	0.00	0.00	0.00	0.00	0.00	0.00	0.00	0.00	3.00	0.00	0.00	7.00	1.00	low
ID203	1.00	3.00	1.00	0.00	0.00	0.00	0.00	0.00	0.00	0.00	0.00	3.00	0.00	0.00	7.00	1.00	low
ID209	1.00	3.00	0.00	0.00	0.00	0.00	0.00	0.00	1.00	0.00	0.00	3.00	0.00	0.00	7.00	1.00	low
ID222	1.00	3.00	1.00	0.00	0.00	0.00	0.00	0.00	0.00	0.00	0.00	3.00	0.00	0.00	7.00	1.00	low
ID224	1.00	3.00	0.00	0.00	1.00	0.00	0.00	0.00	0.00	0.00	0.00	3.00	0.00	0.00	7.00	1.00	low
ID232	2.00	3.00	1.00	0.00	0.00	0.00	0.00	0.00	0.00	0.00	0.00	3.00	0.00	0.00	7.00	1.00	low
ID234	2.00	3.00	1.00	0.00	0.00	0.00	0.00	0.00	0.00	0.00	0.00	3.00	0.00	0.00	7.00	1.00	low
ID235	2.00	3.00	1.00	0.00	0.00	0.00	0.00	0.00	0.00	0.00	3.00	0.00	0.00	0.00	7.00	1.00	low
ID248	2.00	3.00	0.00	0.00	0.00	0.00	1.00	0.00	0.00	0.00	0.00	3.00	0.00	0.00	7.00	1.00	low
ID255	2.00	3.00	1.00	0.00	0.00	0.00	0.00	0.00	0.00	0.00	0.00	3.00	0.00	0.00	7.00	1.00	low
ID38	2.00	3.00	0.00	2.00	0.00	0.00	0.00	0.00	0.00	0.00	0.00	3.00	0.00	0.00	8.00	1.00	low
ID40	2.00	3.00	1.00	0.00	0.00	0.00	1.00	0.00	0.00	0.00	0.00	3.00	0.00	0.00	8.00	1.00	low
ID54	2.00	3.00	1.00	0.00	1.00	0.00	0.00	0.00	0.00	0.00	0.00	3.00	0.00	0.00	8.00	1.00	low
ID61	2.00	3.00	1.00	0.00	1.00	0.00	0.00	0.00	0.00	0.00	0.00	3.00	0.00	0.00	8.00	1.00	low
ID62	2.00	3.00	0.00	0.00	1.00	0.00	0.00	0.00	1.00	0.00	0.00	3.00	0.00	0.00	8.00	1.00	low
ID68	2.00	3.00	1.00	0.00	0.00	0.00	0.00	0.00	1.00	0.00	0.00	3.00	0.00	0.00	8.00	1.00	low
ID73	2.00	3.00	1.00	0.00	0.00	0.00	0.00	0.00	1.00	0.00	0.00	3.00	0.00	0.00	8.00	1.00	low
ID78	2.00	3.00	1.00	0.00	1.00	0.00	0.00	0.00	0.00	3.00	0.00	0.00	0.00	0.00	8.00	1.00	low
ID87	2.00	3.00	1.00	0.00	1.00	0.00	0.00	0.00	0.00	0.00	0.00	3.00	0.00	0.00	8.00	1.00	low
ID92	2.00	3.00	1.00	0.00	0.00	0.00	0.00	0.00	1.00	0.00	0.00	3.00	0.00	0.00	8.00	1.00	low
ID108	2.00	3.00	1.00	0.00	0.00	0.00	0.00	0.00	1.00	0.00	0.00	3.00	0.00	0.00	8.00	1.00	low
ID109	2.00	3.00	1.00	0.00	0.00	0.00	0.00	0.00	1.00	0.00	0.00	3.00	0.00	0.00	8.00	1.00	low
ID117	2.00	3.00	1.00	0.00	1.00	0.00	0.00	0.00	0.00	0.00	0.00	3.00	0.00	0.00	8.00	1.00	low
ID134	2.00	3.00	0.00	0.00	1.00	0.00	0.00	0.00	1.00	0.00	0.00	3.00	0.00	0.00	8.00	1.00	low
ID136	2.00	3.00	1.00	0.00	0.00	0.00	0.00	0.00	1.00	0.00	0.00	3.00	0.00	0.00	8.00	1.00	low
ID138	1.00	3.00	0.00	0.00	0.00	0.00	1.00	0.00	1.00	0.00	0.00	3.00	0.00	0.00	8.00	1.00	low
ID144	1.00	3.00	1.00	0.00	0.00	0.00	1.00	0.00	0.00	0.00	0.00	3.00	0.00	0.00	8.00	1.00	low
ID147	1.00	3.00	1.00	0.00	0.00	0.00	0.00	0.00	1.00	0.00	0.00	3.00	0.00	0.00	8.00	1.00	low
ID149	1.00	3.00	1.00	0.00	0.00	0.00	0.00	0.00	0.00	0.00	0.00	3.00	0.00	1.00	8.00	1.00	low
ID172	1.00	3.00	1.00	0.00	0.00	0.00	0.00	0.00	1.00	0.00	0.00	3.00	0.00	0.00	8.00	1.00	low
ID177	1.00	3.00	1.00	0.00	0.00	0.00	1.00	0.00	0.00	0.00	0.00	3.00	0.00	0.00	8.00	1.00	low
ID185	1.00	3.00	1.00	0.00	0.00	1.00	0.00	0.00	0.00	0.00	0.00	3.00	0.00	0.00	8.00	1.00	low
ID202	1.00	3.00	0.00	0.00	1.00	0.00	0.00	0.00	1.00	0.00	0.00	3.00	0.00	0.00	8.00	1.00	low
ID225	1.00	3.00	1.00	0.00	1.00	0.00	0.00	0.00	0.00	0.00	0.00	3.00	0.00	0.00	8.00	1.00	low
ID226	1.00	3.00	1.00	0.00	0.00	0.00	1.00	0.00	0.00	0.00	0.00	3.00	0.00	0.00	8.00	1.00	low
ID245	2.00	3.00	0.00	0.00	1.00	0.00	0.00	0.00	1.00	0.00	0.00	3.00	0.00	0.00	8.00	1.00	low
ID259	2.00	3.00	1.00	0.00	0.00	0.00	0.00	0.00	1.00	0.00	0.00	3.00	0.00	0.00	8.00	1.00	low
ID7	1.00	3.00	0.00	0.00	0.00	0.00	0.00	0.00	0.00	0.00	3.00	3.00	0.00	0.00	9.00	1.00	low
ID8	1.00	3.00	0.00	0.00	0.00	0.00	0.00	0.00	0.00	0.00	3.00	3.00	0.00	0.00	9.00	1.00	low
ID12	1.00	3.00	1.00	0.00	1.00	0.00	0.00	0.00	1.00	0.00	0.00	3.00	0.00	0.00	9.00	1.00	low
ID35	2.00	3.00	1.00	2.00	0.00	0.00	0.00	0.00	0.00	0.00	0.00	3.00	0.00	0.00	9.00	1.00	low
ID63	2.00	3.00	1.00	0.00	1.00	0.00	0.00	0.00	1.00	0.00	0.00	3.00	0.00	0.00	9.00	1.00	low
ID66	2.00	3.00	1.00	2.00	0.00	0.00	0.00	0.00	0.00	0.00	0.00	3.00	0.00	0.00	9.00	1.00	low
ID77	2.00	3.00	1.00	2.00	0.00	0.00	0.00	0.00	0.00	0.00	3.00	0.00	0.00	0.00	9.00	1.00	low
ID99	2.00	0.00	1.00	0.00	0.00	0.00	0.00	0.00	0.00	0.00	3.00	3.00	2.00	0.00	9.00	1.00	low
ID107	2.00	3.00	1.00	0.00	1.00	0.00	0.00	0.00	1.00	3.00	0.00	0.00	0.00	0.00	9.00	1.00	low
ID129	2.00	3.00	0.00	0.00	1.00	0.00	0.00	0.00	0.00	0.00	0.00	3.00	2.00	0.00	9.00	1.00	low
ID135	2.00	3.00	1.00	0.00	1.00	0.00	0.00	0.00	1.00	0.00	0.00	3.00	0.00	0.00	9.00	1.00	low
ID143	1.00	3.00	1.00	0.00	1.00	0.00	0.00	0.00	1.00	0.00	0.00	3.00	0.00	0.00	9.00	1.00	low
ID161	1.00	3.00	0.00	0.00	0.00	0.00	0.00	0.00	0.00	3.00	0.00	3.00	0.00	0.00	9.00	1.00	low
ID164	1.00	3.00	1.00	0.00	0.00	0.00	0.00	0.00	0.00	0.00	0.00	3.00	2.00	0.00	9.00	1.00	low
ID167	1.00	3.00	0.00	2.00	0.00	1.00	0.00	0.00	0.00	0.00	0.00	3.00	0.00	0.00	9.00	1.00	low
ID183	1.00	3.00	0.00	0.00	1.00	0.00	1.00	0.00	1.00	0.00	0.00	3.00	0.00	0.00	9.00	1.00	low
ID187	1.00	3.00	1.00	0.00	1.00	0.00	0.00	0.00	1.00	0.00	0.00	3.00	0.00	0.00	9.00	1.00	low
ID189	1.00	3.00	0.00	0.00	1.00	0.00	0.00	0.00	0.00	0.00	0.00	3.00	2.00	0.00	9.00	1.00	low
ID191	1.00	3.00	1.00	2.00	0.00	0.00	0.00	0.00	0.00	0.00	0.00	3.00	0.00	0.00	9.00	1.00	low
ID192	1.00	3.00	1.00	0.00	0.00	0.00	0.00	0.00	0.00	0.00	0.00	3.00	2.00	0.00	9.00	1.00	low
ID198	1.00	3.00	1.00	0.00	1.00	0.00	1.00	0.00	0.00	0.00	0.00	3.00	0.00	0.00	9.00	1.00	low
ID205	1.00	3.00	0.00	0.00	0.00	0.00	1.00	0.00	1.00	0.00	0.00	3.00	0.00	1.00	9.00	1.00	low
ID216	1.00	3.00	0.00	2.00	0.00	0.00	0.00	0.00	1.00	0.00	0.00	3.00	0.00	0.00	9.00	1.00	low
ID258	2.00	3.00	0.00	0.00	1.00	0.00	1.00	0.00	1.00	0.00	0.00	3.00	0.00	0.00	9.00	1.00	low
ID262	2.00	3.00	1.00	2.00	0.00	0.00	0.00	0.00	0.00	0.00	0.00	3.00	0.00	0.00	9.00	1.00	low
ID1	1.00	3.00	0.00	0.00	0.00	1.00	0.00	0.00	0.00	0.00	3.00	3.00	0.00	0.00	10.00	1.00	low
ID4	1.00	3.00	0.00	2.00	1.00	0.00	0.00	0.00	1.00	0.00	0.00	3.00	0.00	0.00	10.00	1.00	low
ID13	1.00	3.00	1.00	0.00	0.00	0.00	0.00	0.00	0.00	0.00	3.00	3.00	0.00	0.00	10.00	1.00	low
ID19	1.00	3.00	1.00	0.00	0.00	0.00	0.00	0.00	0.00	0.00	3.00	3.00	0.00	0.00	10.00	1.00	low
ID21	1.00	3.00	0.00	0.00	0.00	0.00	0.00	0.00	1.00	0.00	3.00	3.00	0.00	0.00	10.00	1.00	low
ID27	2.00	3.00	0.00	0.00	0.00	0.00	1.00	0.00	0.00	3.00	0.00	3.00	0.00	0.00	10.00	1.00	low
ID41	2.00	3.00	1.00	0.00	0.00	0.00	0.00	0.00	0.00	3.00	0.00	3.00	0.00	0.00	10.00	1.00	low
ID44	2.00	3.00	0.00	0.00	1.00	0.00	0.00	0.00	0.00	3.00	0.00	3.00	0.00	0.00	10.00	1.00	low
ID53	2.00	3.00	0.00	0.00	0.00	0.00	1.00	0.00	0.00	0.00	3.00	3.00	0.00	0.00	10.00	1.00	low
ID67	2.00	3.00	1.00	2.00	0.00	0.00	0.00	0.00	1.00	0.00	0.00	3.00	0.00	0.00	10.00	1.00	low
ID69	2.00	3.00	0.00	0.00	0.00	0.00	1.00	0.00	0.00	3.00	3.00	0.00	0.00	0.00	10.00	1.00	low
ID79	2.00	3.00	1.00	0.00	0.00	0.00	0.00	0.00	0.00	0.00	3.00	3.00	0.00	0.00	10.00	1.00	low
ID96	2.00	3.00	1.00	0.00	0.00	0.00	0.00	0.00	0.00	3.00	0.00	3.00	0.00	0.00	10.00	1.00	low
ID111	2.00	3.00	0.00	0.00	0.00	0.00	0.00	1.00	0.00	3.00	0.00	3.00	0.00	0.00	10.00	1.00	low
ID121	2.00	3.00	0.00	0.00	0.00	0.00	0.00	0.00	1.00	0.00	3.00	3.00	0.00	0.00	10.00	1.00	low
ID124	2.00	3.00	0.00	0.00	0.00	0.00	0.00	1.00	0.00	0.00	3.00	3.00	0.00	0.00	10.00	1.00	low
ID128	2.00	3.00	0.00	0.00	0.00	0.00	1.00	0.00	0.00	3.00	0.00	3.00	0.00	0.00	10.00	1.00	low
ID131	2.00	3.00	1.00	2.00	1.00	0.00	0.00	0.00	0.00	0.00	0.00	3.00	0.00	0.00	10.00	1.00	low
ID141	1.00	3.00	1.00	2.00	0.00	0.00	0.00	0.00	1.00	0.00	0.00	3.00	0.00	0.00	10.00	1.00	low
ID146	1.00	3.00	0.00	2.00	0.00	1.00	0.00	0.00	1.00	0.00	0.00	3.00	0.00	0.00	10.00	1.00	low
ID150	1.00	0.00	1.00	2.00	0.00	0.00	0.00	0.00	1.00	0.00	3.00	3.00	0.00	0.00	10.00	1.00	low
ID170	1.00	3.00	0.00	0.00	0.00	0.00	0.00	1.00	0.00	0.00	3.00	3.00	0.00	0.00	10.00	1.00	low
ID179	1.00	3.00	1.00	0.00	0.00	0.00	0.00	0.00	0.00	0.00	3.00	3.00	0.00	0.00	10.00	1.00	low
ID190	1.00	3.00	0.00	0.00	0.00	0.00	0.00	0.00	1.00	3.00	0.00	3.00	0.00	0.00	10.00	1.00	low
ID193	1.00	3.00	1.00	2.00	0.00	0.00	0.00	0.00	1.00	0.00	0.00	3.00	0.00	0.00	10.00	1.00	low
ID201	1.00	3.00	1.00	0.00	0.00	0.00	0.00	0.00	0.00	0.00	3.00	3.00	0.00	0.00	10.00	1.00	low
ID206	1.00	3.00	0.00	0.00	0.00	0.00	0.00	1.00	0.00	0.00	3.00	3.00	0.00	0.00	10.00	1.00	low
ID212	1.00	3.00	0.00	0.00	0.00	0.00	0.00	0.00	1.00	3.00	0.00	3.00	0.00	0.00	10.00	1.00	low
ID228	1.00	3.00	0.00	0.00	0.00	1.00	0.00	0.00	0.00	0.00	3.00	3.00	0.00	0.00	10.00	1.00	low
ID229	2.00	3.00	0.00	0.00	0.00	0.00	1.00	1.00	0.00	0.00	0.00	3.00	2.00	0.00	10.00	1.00	low
ID236	2.00	3.00	1.00	2.00	0.00	0.00	1.00	0.00	0.00	0.00	0.00	3.00	0.00	0.00	10.00	1.00	low
ID240	2.00	3.00	0.00	0.00	0.00	0.00	1.00	0.00	0.00	0.00	3.00	3.00	0.00	0.00	10.00	1.00	low
ID241	2.00	3.00	0.00	0.00	1.00	0.00	0.00	0.00	0.00	0.00	3.00	3.00	0.00	0.00	10.00	1.00	low
ID243	2.00	3.00	0.00	0.00	0.00	0.00	1.00	0.00	0.00	0.00	3.00	3.00	0.00	0.00	10.00	1.00	low
ID247	2.00	3.00	1.00	0.00	1.00	0.00	0.00	0.00	0.00	0.00	0.00	3.00	2.00	0.00	10.00	1.00	low
ID250	2.00	3.00	0.00	0.00	0.00	0.00	1.00	0.00	0.00	3.00	0.00	3.00	0.00	0.00	10.00	1.00	low
ID254	2.00	3.00	1.00	2.00	0.00	0.00	1.00	0.00	0.00	0.00	3.00	0.00	0.00	0.00	10.00	1.00	low
ID6	1.00	3.00	1.00	0.00	0.00	0.00	0.00	0.00	1.00	0.00	3.00	3.00	0.00	0.00	11.00	1.00	low
ID9	1.00	3.00	1.00	0.00	0.00	1.00	0.00	0.00	0.00	0.00	3.00	3.00	0.00	0.00	11.00	1.00	low
ID26	1.00	3.00	1.00	0.00	0.00	0.00	0.00	0.00	1.00	0.00	3.00	3.00	0.00	0.00	11.00	1.00	low
ID36	2.00	3.00	1.00	0.00	1.00	0.00	0.00	0.00	0.00	0.00	3.00	3.00	0.00	0.00	11.00	1.00	low
ID39	2.00	3.00	0.00	0.00	0.00	0.00	0.00	0.00	0.00	0.00	3.00	3.00	2.00	0.00	11.00	1.00	low
ID43	2.00	3.00	0.00	0.00	0.00	0.00	0.00	1.00	1.00	3.00	0.00	3.00	0.00	0.00	11.00	1.00	low
ID56	2.00	3.00	1.00	2.00	0.00	0.00	0.00	1.00	1.00	0.00	0.00	3.00	0.00	0.00	11.00	1.00	low
ID57	2.00	3.00	0.00	2.00	1.00	0.00	0.00	0.00	1.00	0.00	0.00	3.00	0.00	1.00	11.00	1.00	low
ID82	2.00	3.00	1.00	0.00	0.00	0.00	0.00	1.00	0.00	0.00	3.00	3.00	0.00	0.00	11.00	1.00	low
ID98	2.00	3.00	1.00	2.00	0.00	0.00	1.00	1.00	0.00	0.00	0.00	3.00	0.00	0.00	11.00	1.00	low
ID112	2.00	3.00	1.00	0.00	0.00	0.00	0.00	0.00	1.00	0.00	3.00	3.00	0.00	0.00	11.00	1.00	low
ID115	2.00	3.00	1.00	0.00	0.00	0.00	0.00	0.00	1.00	0.00	3.00	3.00	0.00	0.00	11.00	1.00	low
ID132	2.00	3.00	1.00	0.00	0.00	0.00	0.00	0.00	1.00	0.00	3.00	3.00	0.00	0.00	11.00	1.00	low
ID162	1.00	3.00	0.00	2.00	0.00	0.00	0.00	0.00	0.00	0.00	3.00	3.00	0.00	0.00	11.00	1.00	low
ID180	1.00	3.00	1.00	0.00	1.00	0.00	0.00	0.00	0.00	0.00	3.00	3.00	0.00	0.00	11.00	1.00	low
ID17	1.00	3.00	0.00	2.00	0.00	0.00	0.00	0.00	1.00	3.00	0.00	3.00	0.00	0.00	12.00	1.00	low
ID25	1.00	3.00	1.00	0.00	1.00	0.00	0.00	0.00	1.00	0.00	3.00	3.00	0.00	0.00	12.00	1.00	low
ID32	2.00	3.00	0.00	0.00	0.00	0.00	0.00	0.00	0.00	3.00	3.00	3.00	0.00	0.00	12.00	1.00	low
ID42	2.00	3.00	0.00	2.00	0.00	1.00	0.00	0.00	0.00	0.00	3.00	3.00	0.00	0.00	12.00	1.00	low
ID45	2.00	3.00	1.00	0.00	0.00	0.00	1.00	1.00	0.00	3.00	3.00	0.00	0.00	0.00	12.00	1.00	low
ID48	2.00	3.00	1.00	0.00	0.00	0.00	0.00	1.00	1.00	0.00	3.00	3.00	0.00	0.00	12.00	1.00	low
ID52	2.00	3.00	1.00	0.00	0.00	0.00	0.00	0.00	0.00	3.00	0.00	3.00	2.00	0.00	12.00	1.00	low
ID58	2.00	3.00	0.00	0.00	0.00	1.00	0.00	1.00	1.00	3.00	0.00	3.00	0.00	0.00	12.00	1.00	low
ID72	2.00	3.00	1.00	0.00	0.00	0.00	1.00	1.00	0.00	0.00	3.00	3.00	0.00	0.00	12.00	1.00	low
ID81	2.00	3.00	0.00	0.00	0.00	0.00	0.00	0.00	0.00	3.00	3.00	3.00	0.00	0.00	12.00	1.00	low
ID90	2.00	3.00	1.00	0.00	1.00	0.00	0.00	0.00	1.00	0.00	3.00	3.00	0.00	0.00	12.00	1.00	low
ID139	1.00	3.00	1.00	0.00	1.00	0.00	1.00	0.00	0.00	0.00	3.00	3.00	0.00	0.00	12.00	1.00	low
ID153	1.00	3.00	1.00	0.00	0.00	1.00	1.00	0.00	0.00	3.00	0.00	3.00	0.00	0.00	12.00	1.00	low
ID155	1.00	3.00	1.00	2.00	0.00	0.00	0.00	0.00	0.00	0.00	3.00	3.00	0.00	0.00	12.00	1.00	low
ID158	1.00	3.00	1.00	0.00	0.00	0.00	0.00	1.00	1.00	0.00	3.00	3.00	0.00	0.00	12.00	1.00	low
ID160	1.00	3.00	0.00	2.00	0.00	0.00	0.00	0.00	1.00	0.00	3.00	3.00	0.00	0.00	12.00	1.00	low
ID166	1.00	3.00	1.00	2.00	0.00	0.00	0.00	0.00	0.00	0.00	3.00	3.00	0.00	0.00	12.00	1.00	low
ID171	1.00	3.00	1.00	0.00	1.00	0.00	0.00	0.00	1.00	3.00	0.00	3.00	0.00	0.00	12.00	1.00	low
ID175	1.00	3.00	0.00	0.00	0.00	0.00	0.00	0.00	0.00	3.00	3.00	3.00	0.00	0.00	12.00	1.00	low
ID176	1.00	3.00	0.00	0.00	0.00	0.00	0.00	0.00	0.00	3.00	3.00	3.00	0.00	0.00	12.00	1.00	low
ID188	1.00	3.00	1.00	0.00	1.00	0.00	0.00	0.00	1.00	0.00	3.00	3.00	0.00	0.00	12.00	1.00	low
ID197	1.00	3.00	1.00	0.00	0.00	0.00	1.00	1.00	0.00	0.00	3.00	3.00	0.00	0.00	12.00	1.00	low
ID208	1.00	3.00	0.00	2.00	1.00	0.00	0.00	0.00	1.00	0.00	0.00	3.00	2.00	0.00	12.00	1.00	low
ID213	1.00	3.00	1.00	2.00	1.00	0.00	0.00	0.00	1.00	0.00	0.00	3.00	0.00	1.00	12.00	1.00	low
ID219	1.00	3.00	0.00	0.00	0.00	1.00	0.00	0.00	0.00	0.00	3.00	3.00	2.00	0.00	12.00	1.00	low
ID239	2.00	3.00	0.00	0.00	0.00	0.00	0.00	0.00	0.00	3.00	3.00	3.00	0.00	0.00	12.00	1.00	low
ID252	2.00	3.00	1.00	2.00	0.00	0.00	0.00	0.00	0.00	0.00	3.00	3.00	0.00	0.00	12.00	1.00	low
ID263	2.00	3.00	1.00	0.00	0.00	0.00	1.00	1.00	1.00	0.00	0.00	3.00	2.00	0.00	12.00	1.00	low
ID265	2.00	3.00	1.00	2.00	0.00	0.00	0.00	0.00	0.00	0.00	3.00	3.00	0.00	0.00	12.00	1.00	low
ID2	1.00	3.00	1.00	0.00	0.00	0.00	1.00	1.00	1.00	0.00	3.00	3.00	0.00	0.00	13.00	2.00	high
ID20	1.00	3.00	1.00	0.00	0.00	0.00	0.00	0.00	1.00	0.00	3.00	3.00	2.00	0.00	13.00	2.00	high
ID22	1.00	3.00	0.00	0.00	0.00	0.00	0.00	0.00	1.00	3.00	3.00	3.00	0.00	0.00	13.00	2.00	high
ID23	1.00	3.00	0.00	0.00	0.00	0.00	0.00	0.00	1.00	3.00	3.00	3.00	0.00	0.00	13.00	2.00	high
ID24	1.00	3.00	0.00	0.00	0.00	0.00	1.00	0.00	0.00	3.00	3.00	3.00	0.00	0.00	13.00	2.00	high
ID51	2.00	3.00	1.00	0.00	0.00	0.00	1.00	0.00	1.00	0.00	3.00	3.00	0.00	1.00	13.00	2.00	high
ID59	2.00	3.00	1.00	0.00	0.00	0.00	0.00	0.00	0.00	3.00	3.00	3.00	0.00	0.00	13.00	2.00	high
ID83	2.00	3.00	1.00	0.00	1.00	0.00	0.00	0.00	0.00	0.00	3.00	3.00	2.00	0.00	13.00	2.00	high
ID86	2.00	3.00	1.00	0.00	0.00	0.00	0.00	0.00	0.00	3.00	3.00	3.00	0.00	0.00	13.00	2.00	high
ID88	2.00	3.00	0.00	0.00	0.00	0.00	1.00	0.00	0.00	3.00	3.00	3.00	0.00	0.00	13.00	2.00	high
ID91	2.00	3.00	0.00	0.00	1.00	0.00	0.00	0.00	0.00	3.00	3.00	3.00	0.00	0.00	13.00	2.00	high
ID130	2.00	3.00	1.00	0.00	0.00	1.00	1.00	0.00	1.00	0.00	3.00	3.00	0.00	0.00	13.00	2.00	high
ID182	1.00	3.00	1.00	0.00	0.00	0.00	0.00	0.00	0.00	3.00	3.00	3.00	0.00	0.00	13.00	2.00	high
ID204	1.00	3.00	1.00	0.00	1.00	0.00	0.00	0.00	0.00	0.00	3.00	3.00	2.00	0.00	13.00	2.00	high
ID214	1.00	3.00	1.00	0.00	1.00	1.00	1.00	0.00	1.00	0.00	0.00	3.00	2.00	0.00	13.00	2.00	high
ID215	1.00	3.00	1.00	2.00	1.00	0.00	0.00	0.00	0.00	0.00	3.00	3.00	0.00	0.00	13.00	2.00	high
ID227	1.00	3.00	1.00	0.00	1.00	0.00	1.00	0.00	1.00	3.00	0.00	3.00	0.00	0.00	13.00	2.00	high
ID230	2.00	3.00	0.00	0.00	0.00	0.00	1.00	0.00	0.00	3.00	3.00	3.00	0.00	0.00	13.00	2.00	high
ID233	2.00	3.00	1.00	0.00	0.00	0.00	0.00	0.00	1.00	0.00	3.00	3.00	2.00	0.00	13.00	2.00	high
ID246	2.00	3.00	0.00	0.00	0.00	0.00	1.00	0.00	0.00	3.00	3.00	3.00	0.00	0.00	13.00	2.00	high
ID256	2.00	3.00	1.00	2.00	1.00	0.00	0.00	0.00	0.00	3.00	0.00	3.00	0.00	0.00	13.00	2.00	high
ID10	1.00	3.00	0.00	0.00	0.00	0.00	1.00	1.00	0.00	3.00	3.00	3.00	0.00	0.00	14.00	2.00	high
ID16	1.00	3.00	1.00	0.00	0.00	0.00	0.00	1.00	0.00	3.00	3.00	3.00	0.00	0.00	14.00	2.00	high
ID29	2.00	3.00	1.00	0.00	1.00	0.00	0.00	0.00	0.00	3.00	3.00	3.00	0.00	0.00	14.00	2.00	high
ID31	2.00	3.00	0.00	0.00	0.00	0.00	1.00	1.00	0.00	3.00	3.00	3.00	0.00	0.00	14.00	2.00	high
ID34	2.00	3.00	0.00	0.00	1.00	0.00	0.00	1.00	0.00	3.00	3.00	3.00	0.00	0.00	14.00	2.00	high
ID65	2.00	3.00	1.00	0.00	0.00	0.00	1.00	0.00	0.00	3.00	3.00	3.00	0.00	0.00	14.00	2.00	high
ID84	2.00	3.00	1.00	0.00	1.00	0.00	0.00	0.00	0.00	3.00	3.00	3.00	0.00	0.00	14.00	2.00	high
ID101	2.00	3.00	1.00	0.00	0.00	0.00	0.00	1.00	0.00	3.00	3.00	3.00	0.00	0.00	14.00	2.00	high
ID104	2.00	3.00	0.00	0.00	1.00	0.00	0.00	0.00	1.00	3.00	3.00	3.00	0.00	0.00	14.00	2.00	high
ID110	2.00	3.00	0.00	2.00	0.00	0.00	0.00	0.00	1.00	0.00	3.00	3.00	2.00	0.00	14.00	2.00	high
ID113	2.00	3.00	1.00	0.00	0.00	0.00	0.00	0.00	1.00	3.00	3.00	3.00	0.00	0.00	14.00	2.00	high
ID119	2.00	3.00	0.00	0.00	0.00	1.00	0.00	0.00	1.00	3.00	3.00	3.00	0.00	0.00	14.00	2.00	high
ID122	2.00	3.00	0.00	0.00	0.00	0.00	1.00	0.00	1.00	3.00	3.00	3.00	0.00	0.00	14.00	2.00	high
ID152	1.00	3.00	1.00	0.00	1.00	0.00	0.00	0.00	0.00	3.00	3.00	3.00	0.00	0.00	14.00	2.00	high
ID154	1.00	3.00	1.00	0.00	0.00	0.00	0.00	0.00	1.00	3.00	3.00	3.00	0.00	0.00	14.00	2.00	high
ID165	1.00	3.00	1.00	2.00	0.00	0.00	1.00	0.00	1.00	3.00	0.00	3.00	0.00	0.00	14.00	2.00	high
ID217	1.00	3.00	1.00	2.00	1.00	0.00	0.00	0.00	1.00	0.00	3.00	3.00	0.00	0.00	14.00	2.00	high
ID218	1.00	3.00	0.00	0.00	0.00	0.00	1.00	1.00	0.00	3.00	3.00	3.00	0.00	0.00	14.00	2.00	high
ID220	1.00	3.00	1.00	0.00	0.00	0.00	0.00	0.00	1.00	3.00	3.00	3.00	0.00	0.00	14.00	2.00	high
ID253	2.00	3.00	1.00	2.00	0.00	0.00	0.00	1.00	1.00	3.00	0.00	3.00	0.00	0.00	14.00	2.00	high
ID264	2.00	3.00	0.00	2.00	0.00	1.00	0.00	1.00	1.00	0.00	3.00	3.00	0.00	0.00	14.00	2.00	high
ID11	1.00	3.00	0.00	2.00	0.00	0.00	0.00	0.00	1.00	3.00	3.00	3.00	0.00	0.00	15.00	2.00	high
ID15	1.00	3.00	1.00	0.00	0.00	0.00	0.00	1.00	1.00	3.00	3.00	3.00	0.00	0.00	15.00	2.00	high
ID28	2.00	3.00	1.00	2.00	0.00	0.00	0.00	0.00	0.00	3.00	3.00	3.00	0.00	0.00	15.00	2.00	high
ID33	2.00	3.00	1.00	2.00	0.00	0.00	0.00	0.00	0.00	0.00	3.00	3.00	2.00	1.00	15.00	2.00	high
ID37	2.00	3.00	1.00	0.00	0.00	1.00	0.00	0.00	1.00	3.00	3.00	3.00	0.00	0.00	15.00	2.00	high
ID75	2.00	3.00	1.00	0.00	0.00	0.00	1.00	1.00	0.00	3.00	3.00	3.00	0.00	0.00	15.00	2.00	high
ID85	2.00	3.00	1.00	0.00	1.00	0.00	1.00	0.00	0.00	3.00	3.00	3.00	0.00	0.00	15.00	2.00	high
ID116	2.00	3.00	0.00	0.00	1.00	0.00	1.00	0.00	1.00	3.00	3.00	3.00	0.00	0.00	15.00	2.00	high
ID151	1.00	3.00	1.00	0.00	0.00	0.00	0.00	1.00	1.00	3.00	3.00	3.00	0.00	0.00	15.00	2.00	high
ID157	1.00	3.00	0.00	0.00	0.00	1.00	0.00	1.00	1.00	3.00	3.00	3.00	0.00	0.00	15.00	2.00	high
ID173	1.00	3.00	1.00	0.00	0.00	0.00	1.00	0.00	1.00	3.00	3.00	3.00	0.00	0.00	15.00	2.00	high
ID174	1.00	3.00	0.00	0.00	0.00	1.00	0.00	0.00	1.00	3.00	3.00	3.00	0.00	1.00	15.00	2.00	high
ID181	1.00	3.00	1.00	2.00	0.00	0.00	1.00	0.00	0.00	0.00	3.00	3.00	2.00	0.00	15.00	2.00	high
ID200	1.00	3.00	0.00	2.00	1.00	0.00	0.00	0.00	0.00	3.00	3.00	3.00	0.00	0.00	15.00	2.00	high
ID207	1.00	3.00	1.00	2.00	0.00	0.00	0.00	0.00	0.00	3.00	3.00	3.00	0.00	0.00	15.00	2.00	high
ID221	1.00	3.00	1.00	0.00	1.00	0.00	0.00	0.00	1.00	3.00	3.00	3.00	0.00	0.00	15.00	2.00	high
ID261	2.00	3.00	1.00	0.00	1.00	0.00	1.00	0.00	0.00	3.00	3.00	3.00	0.00	0.00	15.00	2.00	high
ID89	2.00	3.00	1.00	2.00	0.00	0.00	1.00	0.00	0.00	3.00	3.00	3.00	0.00	0.00	16.00	2.00	high
ID140	1.00	3.00	1.00	2.00	1.00	0.00	0.00	0.00	1.00	0.00	3.00	3.00	2.00	0.00	16.00	2.00	high
ID148	1.00	3.00	1.00	2.00	0.00	0.00	0.00	0.00	1.00	3.00	3.00	3.00	0.00	0.00	16.00	2.00	high
ID159	1.00	3.00	1.00	0.00	1.00	0.00	0.00	0.00	0.00	3.00	3.00	3.00	2.00	0.00	16.00	2.00	high
ID169	1.00	3.00	0.00	2.00	1.00	0.00	0.00	0.00	1.00	3.00	3.00	3.00	0.00	0.00	16.00	2.00	high
ID184	1.00	3.00	0.00	2.00	0.00	1.00	0.00	0.00	1.00	3.00	3.00	3.00	0.00	0.00	16.00	2.00	high
ID211	1.00	3.00	1.00	0.00	1.00	0.00	0.00	1.00	1.00	3.00	3.00	3.00	0.00	0.00	16.00	2.00	high
ID223	1.00	3.00	1.00	2.00	0.00	0.00	1.00	0.00	0.00	3.00	3.00	3.00	0.00	0.00	16.00	2.00	high
ID231	2.00	3.00	1.00	2.00	0.00	0.00	1.00	0.00	0.00	3.00	3.00	3.00	0.00	0.00	16.00	2.00	high
ID237	2.00	3.00	1.00	2.00	0.00	0.00	1.00	0.00	0.00	3.00	3.00	3.00	0.00	0.00	16.00	2.00	high
ID238	2.00	3.00	1.00	0.00	1.00	0.00	0.00	0.00	0.00	3.00	3.00	3.00	2.00	0.00	16.00	2.00	high
ID244	2.00	3.00	1.00	0.00	0.00	1.00	1.00	1.00	0.00	3.00	3.00	3.00	0.00	0.00	16.00	2.00	high
ID76	2.00	3.00	1.00	2.00	1.00	0.00	0.00	0.00	1.00	3.00	3.00	3.00	0.00	0.00	17.00	2.00	high
ID260	2.00	3.00	1.00	0.00	0.00	0.00	1.00	1.00	0.00	3.00	3.00	3.00	2.00	0.00	17.00	2.00	high
ID105	2.00	3.00	1.00	2.00	0.00	1.00	0.00	1.00	0.00	3.00	3.00	3.00	0.00	0.00	17.00	2.00	high
average score															10.35		

**Table A2:** MDS-preg score calculation according to specific food categories.

ID	Location	Whole Grain	Dairy	Red_Meat	Fish	Legumes	Olive	Vegetables	Fruit	Nuts	Score	Category *	Adherence
ID4	1.00	0.00	0.00	0.00	0.00	0.00	0.00	0.00	0.00	0.00	0.00	1.00	low
ID26	1.00	0.00	0.00	0.00	0.00	0.00	0.00	0.00	0.00	0.00	0.00	1.00	low
ID235	2.00	0.00	0.00	0.00	0.00	0.00	0.00	0.00	0.00	0.00	0.00	1.00	low
ID3	1.00	0.00	0.00	0.00	0.00	0.00	0.00	0.00	1.00	0.00	1.00	1.00	low
ID12	1.00	1.00	0.00	0.00	0.00	0.00	0.00	0.00	0.00	0.00	1.00	1.00	low
ID13	1.00	1.00	0.00	0.00	0.00	0.00	0.00	0.00	0.00	0.00	1.00	1.00	low
ID14	1.00	0.00	0.00	0.00	0.00	0.00	0.00	0.00	0.00	1.00	1.00	1.00	low
ID38	2.00	1.00	0.00	0.00	0.00	0.00	0.00	0.00	0.00	0.00	1.00	1.00	low
ID49	2.00	1.00	0.00	0.00	0.00	0.00	0.00	0.00	0.00	0.00	1.00	1.00	low
ID68	2.00	1.00	0.00	0.00	0.00	0.00	0.00	0.00	0.00	0.00	1.00	1.00	low
ID80	2.00	0.00	0.00	0.00	0.00	0.00	0.00	0.00	1.00	0.00	1.00	1.00	low
ID87	2.00	0.00	0.00	0.00	0.00	0.00	0.00	0.00	1.00	0.00	1.00	1.00	low
ID93	2.00	0.00	0.00	1.00	0.00	0.00	0.00	0.00	0.00	0.00	1.00	1.00	low
ID96	2.00	0.00	0.00	0.00	0.00	0.00	1.00	0.00	0.00	0.00	1.00	1.00	low
ID97	2.00	0.00	0.00	0.00	0.00	0.00	0.00	0.00	1.00	0.00	1.00	1.00	low
ID108	2.00	0.00	0.00	1.00	0.00	0.00	0.00	0.00	0.00	0.00	1.00	1.00	low
ID112	2.00	0.00	0.00	0.00	0.00	0.00	0.00	1.00	0.00	0.00	1.00	1.00	low
ID127	2.00	0.00	0.00	0.00	1.00	0.00	0.00	0.00	0.00	0.00	1.00	1.00	low
ID142	2.00	0.00	0.00	0.00	0.00	1.00	0.00	0.00	0.00	0.00	1.00	1.00	low
ID156	2.00	0.00	0.00	0.00	1.00	0.00	0.00	0.00	0.00	0.00	1.00	1.00	low
ID168	1.00	1.00	0.00	0.00	0.00	0.00	0.00	0.00	0.00	0.00	1.00	1.00	low
ID186	1.00	0.00	0.00	1.00	0.00	0.00	0.00	0.00	0.00	0.00	1.00	1.00	low
ID195	1.00	1.00	0.00	0.00	0.00	0.00	0.00	0.00	0.00	0.00	1.00	1.00	low
ID202	1.00	1.00	0.00	0.00	0.00	0.00	0.00	0.00	0.00	0.00	1.00	1.00	low
ID203	1.00	0.00	0.00	0.00	0.00	0.00	0.00	0.00	0.00	1.00	1.00	1.00	low
ID217	1.00	1.00	0.00	0.00	0.00	0.00	0.00	0.00	0.00	0.00	1.00	1.00	low
ID251	2.00	1.00	0.00	0.00	0.00	0.00	0.00	0.00	0.00	0.00	1.00	1.00	low
ID259	2.00	1.00	0.00	0.00	0.00	0.00	0.00	0.00	0.00	0.00	1.00	1.00	low
ID266	2.00	1.00	0.00	0.00	0.00	0.00	0.00	0.00	0.00	0.00	1.00	1.00	low
ID7	1.00	0.00	0.00	0.00	0.00	0.00	0.00	0.00	1.00	1.00	2.00	1.00	low
ID27	2.00	0.00	0.00	0.00	1.00	0.00	1.00	0.00	0.00	0.00	2.00	1.00	low
ID40	2.00	0.00	1.00	0.00	1.00	0.00	0.00	0.00	0.00	0.00	2.00	1.00	low
ID48	2.00	1.00	0.00	0.00	0.00	1.00	0.00	0.00	0.00	0.00	2.00	1.00	low
ID50	2.00	1.00	0.00	0.00	0.00	0.00	0.00	0.00	1.00	0.00	2.00	1.00	low
ID54	2.00	1.00	1.00	0.00	0.00	0.00	0.00	0.00	0.00	0.00	2.00	1.00	low
ID62	2.00	1.00	0.00	1.00	0.00	0.00	0.00	0.00	0.00	0.00	2.00	1.00	low
ID66	2.00	1.00	1.00	0.00	0.00	0.00	0.00	0.00	0.00	0.00	2.00	1.00	low
ID78	2.00	0.00	0.00	1.00	0.00	0.00	1.00	0.00	0.00	0.00	2.00	1.00	low
ID90	2.00	1.00	0.00	0.00	0.00	0.00	0.00	0.00	1.00	0.00	2.00	1.00	low
ID92	2.00	1.00	1.00	0.00	0.00	0.00	0.00	0.00	0.00	0.00	2.00	1.00	low
ID103	2.00	1.00	0.00	0.00	0.00	0.00	0.00	0.00	1.00	0.00	2.00	1.00	low
ID114	2.00	0.00	0.00	0.00	0.00	0.00	0.00	0.00	1.00	1.00	2.00	1.00	low
ID117	2.00	1.00	0.00	1.00	0.00	0.00	0.00	0.00	0.00	0.00	2.00	1.00	low
ID118	2.00	1.00	0.00	0.00	0.00	0.00	0.00	0.00	0.00	1.00	2.00	1.00	low
ID119	2.00	0.00	0.00	1.00	0.00	0.00	1.00	0.00	0.00	0.00	2.00	1.00	low
ID125	2.00	1.00	1.00	0.00	0.00	0.00	0.00	0.00	0.00	0.00	2.00	1.00	low
ID133	2.00	1.00	0.00	1.00	0.00	0.00	0.00	0.00	0.00	0.00	2.00	1.00	low
ID135	2.00	1.00	0.00	0.00	0.00	0.00	0.00	0.00	1.00	0.00	2.00	1.00	low
ID145	2.00	0.00	0.00	1.00	0.00	1.00	0.00	0.00	0.00	0.00	2.00	1.00	low
ID146	2.00	0.00	1.00	1.00	0.00	0.00	0.00	0.00	0.00	0.00	2.00	1.00	low
ID147	2.00	1.00	0.00	0.00	0.00	0.00	0.00	0.00	1.00	0.00	2.00	1.00	low
ID149	2.00	1.00	0.00	0.00	0.00	0.00	0.00	0.00	1.00	0.00	2.00	1.00	low
ID171	1.00	1.00	0.00	0.00	0.00	0.00	1.00	0.00	0.00	0.00	2.00	1.00	low
ID177	1.00	1.00	0.00	0.00	1.00	0.00	0.00	0.00	0.00	0.00	2.00	1.00	low
ID178	1.00	1.00	1.00	0.00	0.00	0.00	0.00	0.00	0.00	0.00	2.00	1.00	low
ID194	1.00	1.00	1.00	0.00	0.00	0.00	0.00	0.00	0.00	0.00	2.00	1.00	low
ID209	1.00	1.00	1.00	0.00	0.00	0.00	0.00	0.00	0.00	0.00	2.00	1.00	low
ID210	1.00	1.00	0.00	1.00	0.00	0.00	0.00	0.00	0.00	0.00	2.00	1.00	low
ID213	1.00	1.00	0.00	0.00	0.00	0.00	0.00	0.00	1.00	0.00	2.00	1.00	low
ID222	1.00	1.00	0.00	0.00	0.00	0.00	0.00	0.00	0.00	1.00	2.00	1.00	low
ID224	1.00	1.00	0.00	0.00	0.00	0.00	0.00	0.00	1.00	0.00	2.00	1.00	low
ID225	1.00	1.00	1.00	0.00	0.00	0.00	0.00	0.00	0.00	0.00	2.00	1.00	low
ID230	2.00	0.00	0.00	0.00	1.00	0.00	1.00	0.00	0.00	0.00	2.00	1.00	low
ID233	2.00	0.00	0.00	0.00	0.00	0.00	0.00	0.00	1.00	1.00	2.00	1.00	low
ID241	2.00	0.00	0.00	0.00	0.00	0.00	0.00	0.00	1.00	1.00	2.00	1.00	low
ID242	2.00	1.00	0.00	0.00	0.00	0.00	0.00	0.00	0.00	1.00	2.00	1.00	low
ID245	2.00	1.00	0.00	1.00	0.00	0.00	0.00	0.00	0.00	0.00	2.00	1.00	low
ID248	2.00	0.00	0.00	0.00	1.00	0.00	0.00	0.00	1.00	0.00	2.00	1.00	low
ID254	2.00	0.00	0.00	1.00	1.00	0.00	0.00	0.00	0.00	0.00	2.00	1.00	low
ID255	2.00	1.00	0.00	0.00	0.00	0.00	0.00	0.00	0.00	1.00	2.00	1.00	low
ID256	2.00	1.00	0.00	0.00	0.00	0.00	1.00	0.00	0.00	0.00	2.00	1.00	low
ID257	2.00	1.00	0.00	1.00	0.00	0.00	0.00	0.00	0.00	0.00	2.00	1.00	low
ID262	2.00	1.00	0.00	0.00	0.00	0.00	0.00	0.00	1.00	0.00	2.00	1.00	low
ID8	1.00	1.00	0.00	1.00	0.00	0.00	0.00	0.00	1.00	0.00	3.00	1.00	low
ID18	1.00	0.00	0.00	1.00	1.00	0.00	0.00	0.00	1.00	0.00	3.00	1.00	low
ID22	1.00	1.00	0.00	1.00	0.00	0.00	1.00	0.00	0.00	0.00	3.00	1.00	low
ID25	1.00	1.00	0.00	1.00	0.00	0.00	0.00	0.00	1.00	0.00	3.00	1.00	low
ID35	2.00	1.00	1.00	0.00	0.00	0.00	0.00	0.00	0.00	1.00	3.00	1.00	low
ID44	2.00	1.00	0.00	1.00	0.00	0.00	1.00	0.00	0.00	0.00	3.00	1.00	low
ID57	2.00	0.00	1.00	1.00	0.00	0.00	0.00	0.00	0.00	1.00	3.00	1.00	low
ID60	2.00	1.00	1.00	0.00	1.00	0.00	0.00	0.00	0.00	0.00	3.00	1.00	low
ID61	2.00	1.00	1.00	0.00	0.00	0.00	0.00	0.00	0.00	1.00	3.00	1.00	low
ID64	2.00	1.00	0.00	0.00	0.00	0.00	0.00	0.00	1.00	1.00	3.00	1.00	low
ID71	2.00	1.00	1.00	0.00	0.00	0.00	0.00	0.00	1.00	0.00	3.00	1.00	low
ID73	2.00	1.00	0.00	1.00	0.00	0.00	0.00	0.00	0.00	1.00	3.00	1.00	low
ID74	2.00	1.00	1.00	0.00	1.00	0.00	0.00	0.00	0.00	0.00	3.00	1.00	low
ID77	2.00	1.00	1.00	0.00	0.00	0.00	0.00	0.00	0.00	1.00	3.00	1.00	low
ID83	2.00	1.00	1.00	0.00	0.00	0.00	0.00	0.00	0.00	1.00	3.00	1.00	low
ID84	2.00	1.00	0.00	0.00	0.00	0.00	1.00	0.00	1.00	0.00	3.00	1.00	low
ID94	2.00	1.00	0.00	1.00	0.00	0.00	0.00	0.00	0.00	1.00	3.00	1.00	low
ID95	2.00	1.00	1.00	0.00	0.00	0.00	0.00	0.00	1.00	0.00	3.00	1.00	low
ID100	2.00	0.00	1.00	0.00	0.00	0.00	0.00	0.00	1.00	1.00	3.00	1.00	low
ID102	2.00	1.00	1.00	0.00	0.00	0.00	0.00	0.00	1.00	0.00	3.00	1.00	low
ID104	2.00	1.00	0.00	0.00	0.00	0.00	1.00	0.00	0.00	1.00	3.00	1.00	low
ID106	2.00	0.00	0.00	1.00	1.00	0.00	0.00	0.00	1.00	0.00	3.00	1.00	low
ID109	2.00	1.00	0.00	1.00	0.00	0.00	0.00	0.00	1.00	0.00	3.00	1.00	low
ID115	2.00	0.00	0.00	1.00	0.00	0.00	0.00	0.00	1.00	1.00	3.00	1.00	low
ID120	2.00	1.00	1.00	0.00	0.00	0.00	0.00	0.00	1.00	0.00	3.00	1.00	low
ID121	2.00	1.00	1.00	0.00	0.00	0.00	0.00	0.00	1.00	0.00	3.00	1.00	low
ID123	2.00	1.00	1.00	0.00	0.00	0.00	0.00	0.00	1.00	0.00	3.00	1.00	low
ID124	2.00	1.00	0.00	1.00	0.00	1.00	0.00	0.00	0.00	0.00	3.00	1.00	low
ID126	2.00	1.00	0.00	1.00	0.00	0.00	0.00	0.00	1.00	0.00	3.00	1.00	low
ID129	2.00	1.00	1.00	0.00	0.00	0.00	0.00	0.00	0.00	1.00	3.00	1.00	low
ID131	2.00	1.00	1.00	0.00	0.00	0.00	0.00	0.00	0.00	1.00	3.00	1.00	low
ID136	2.00	1.00	0.00	1.00	0.00	0.00	0.00	0.00	1.00	0.00	3.00	1.00	low
ID141	2.00	1.00	0.00	0.00	0.00	0.00	0.00	0.00	1.00	1.00	3.00	1.00	low
ID150	2.00	0.00	0.00	1.00	0.00	0.00	0.00	0.00	1.00	1.00	3.00	1.00	low
ID155	2.00	1.00	1.00	0.00	0.00	0.00	0.00	0.00	1.00	0.00	3.00	1.00	low
ID164	1.00	1.00	0.00	0.00	0.00	0.00	0.00	0.00	1.00	1.00	3.00	1.00	low
ID166	1.00	1.00	1.00	0.00	0.00	0.00	0.00	0.00	0.00	1.00	3.00	1.00	low
ID176	1.00	1.00	1.00	0.00	0.00	0.00	1.00	0.00	0.00	0.00	3.00	1.00	low
ID180	1.00	1.00	0.00	1.00	0.00	0.00	0.00	0.00	0.00	1.00	3.00	1.00	low
ID182	1.00	0.00	1.00	0.00	0.00	0.00	1.00	0.00	1.00	0.00	3.00	1.00	low
ID183	1.00	1.00	0.00	0.00	1.00	0.00	0.00	0.00	1.00	0.00	3.00	1.00	low
ID187	1.00	1.00	0.00	0.00	0.00	0.00	0.00	0.00	1.00	1.00	3.00	1.00	low
ID189	1.00	1.00	1.00	0.00	0.00	0.00	0.00	0.00	0.00	1.00	3.00	1.00	low
ID191	1.00	1.00	1.00	0.00	0.00	0.00	0.00	0.00	0.00	1.00	3.00	1.00	low
ID192	1.00	0.00	1.00	1.00	0.00	0.00	0.00	0.00	0.00	1.00	3.00	1.00	low
ID196	1.00	1.00	0.00	1.00	0.00	0.00	0.00	0.00	1.00	0.00	3.00	1.00	low
ID201	1.00	1.00	0.00	0.00	0.00	0.00	0.00	0.00	1.00	1.00	3.00	1.00	low
ID204	1.00	1.00	1.00	0.00	0.00	0.00	0.00	0.00	0.00	1.00	3.00	1.00	low
ID208	1.00	0.00	1.00	0.00	0.00	0.00	0.00	0.00	1.00	1.00	3.00	1.00	low
ID215	1.00	0.00	0.00	0.00	0.00	1.00	0.00	0.00	1.00	1.00	3.00	1.00	low
ID216	1.00	1.00	0.00	0.00	0.00	0.00	0.00	0.00	1.00	1.00	3.00	1.00	low
ID221	1.00	0.00	0.00	0.00	1.00	0.00	1.00	0.00	0.00	1.00	3.00	1.00	low
ID226	1.00	1.00	0.00	0.00	1.00	0.00	0.00	0.00	1.00	0.00	3.00	1.00	low
ID234	2.00	1.00	0.00	0.00	0.00	0.00	0.00	0.00	1.00	1.00	3.00	1.00	low
ID249	2.00	1.00	1.00	1.00	0.00	0.00	0.00	0.00	0.00	0.00	3.00	1.00	low
ID265	2.00	1.00	0.00	0.00	0.00	0.00	0.00	1.00	1.00	0.00	3.00	1.00	low
ID1	1.00	1.00	0.00	1.00	0.00	0.00	0.00	1.00	1.00	0.00	4.00	2.00	mod.
ID5	1.00	1.00	1.00	0.00	0.00	0.00	0.00	0.00	1.00	1.00	4.00	2.00	mod.
ID17	1.00	1.00	0.00	0.00	0.00	0.00	1.00	0.00	1.00	1.00	4.00	2.00	mod.
ID19	1.00	1.00	1.00	0.00	0.00	0.00	0.00	1.00	1.00	0.00	4.00	2.00	mod.
ID23	1.00	1.00	0.00	1.00	0.00	0.00	1.00	0.00	1.00	0.00	4.00	2.00	mod.
ID28	2.00	1.00	0.00	0.00	0.00	0.00	1.00	0.00	1.00	1.00	4.00	2.00	mod.
ID33	2.00	0.00	1.00	0.00	0.00	0.00	0.00	1.00	1.00	1.00	4.00	2.00	mod.
ID36	2.00	1.00	1.00	0.00	0.00	0.00	0.00	0.00	1.00	1.00	4.00	2.00	mod.
ID37	2.00	0.00	1.00	1.00	0.00	0.00	1.00	0.00	1.00	0.00	4.00	2.00	mod.
ID41	2.00	1.00	1.00	0.00	0.00	0.00	1.00	0.00	1.00	0.00	4.00	2.00	mod.
ID46	2.00	1.00	1.00	1.00	0.00	0.00	0.00	0.00	1.00	0.00	4.00	2.00	mod.
ID47	2.00	1.00	1.00	0.00	0.00	0.00	0.00	0.00	1.00	1.00	4.00	2.00	mod.
ID53	2.00	1.00	1.00	0.00	1.00	0.00	0.00	0.00	1.00	0.00	4.00	2.00	mod.
ID55	2.00	1.00	1.00	1.00	0.00	0.00	0.00	0.00	1.00	0.00	4.00	2.00	mod.
ID56	2.00	0.00	1.00	1.00	0.00	1.00	0.00	0.00	1.00	0.00	4.00	2.00	mod.
ID58	2.00	1.00	0.00	1.00	0.00	1.00	1.00	0.00	0.00	0.00	4.00	2.00	mod.
ID63	2.00	1.00	1.00	0.00	1.00	0.00	0.00	0.00	0.00	1.00	4.00	2.00	mod.
ID67	2.00	1.00	0.00	1.00	1.00	0.00	0.00	0.00	0.00	1.00	4.00	2.00	mod.
ID70	2.00	1.00	0.00	1.00	0.00	0.00	0.00	0.00	1.00	1.00	4.00	2.00	mod.
ID76	2.00	1.00	0.00	0.00	0.00	0.00	1.00	1.00	1.00	0.00	4.00	2.00	mod.
ID81	2.00	1.00	1.00	0.00	0.00	0.00	1.00	0.00	1.00	0.00	4.00	2.00	mod.
ID98	2.00	1.00	1.00	0.00	1.00	1.00	0.00	0.00	0.00	0.00	4.00	2.00	mod.
ID107	2.00	1.00	1.00	0.00	0.00	0.00	1.00	0.00	0.00	1.00	4.00	2.00	mod.
ID132	2.00	1.00	0.00	1.00	0.00	0.00	0.00	1.00	0.00	1.00	4.00	2.00	mod.
ID134	2.00	1.00	1.00	1.00	0.00	0.00	0.00	0.00	1.00	0.00	4.00	2.00	mod.
ID137	2.00	1.00	1.00	1.00	0.00	0.00	0.00	0.00	0.00	1.00	4.00	2.00	mod.
ID138	2.00	1.00	0.00	0.00	1.00	0.00	0.00	0.00	1.00	1.00	4.00	2.00	mod.
ID139	2.00	1.00	1.00	0.00	1.00	0.00	0.00	0.00	1.00	0.00	4.00	2.00	mod.
ID140	2.00	1.00	0.00	1.00	0.00	0.00	0.00	0.00	1.00	1.00	4.00	2.00	mod.
ID143	2.00	1.00	1.00	1.00	0.00	0.00	0.00	0.00	0.00	1.00	4.00	2.00	mod.
ID144	2.00	1.00	0.00	1.00	1.00	0.00	0.00	0.00	1.00	0.00	4.00	2.00	mod.
ID148	2.00	1.00	0.00	0.00	0.00	0.00	1.00	1.00	1.00	0.00	4.00	2.00	mod.
ID152	2.00	1.00	0.00	1.00	0.00	0.00	1.00	0.00	0.00	1.00	4.00	2.00	mod.
ID158	2.00	1.00	0.00	1.00	0.00	1.00	0.00	0.00	1.00	0.00	4.00	2.00	mod.
ID160	2.00	0.00	1.00	1.00	0.00	0.00	0.00	0.00	1.00	1.00	4.00	2.00	mod.
ID161	1.00	1.00	0.00	1.00	0.00	0.00	1.00	0.00	1.00	0.00	4.00	2.00	mod.
ID162	1.00	1.00	1.00	0.00	0.00	0.00	0.00	1.00	1.00	0.00	4.00	2.00	mod.
ID163	1.00	1.00	1.00	0.00	0.00	0.00	0.00	0.00	1.00	1.00	4.00	2.00	mod.
ID167	1.00	1.00	0.00	1.00	0.00	0.00	0.00	0.00	1.00	1.00	4.00	2.00	mod.
ID169	1.00	1.00	1.00	0.00	0.00	0.00	1.00	1.00	0.00	0.00	4.00	2.00	mod.
ID170	1.00	1.00	1.00	0.00	0.00	1.00	0.00	0.00	0.00	1.00	4.00	2.00	mod.
ID172	1.00	1.00	1.00	1.00	0.00	0.00	0.00	0.00	1.00	0.00	4.00	2.00	mod.
ID175	1.00	1.00	0.00	0.00	0.00	0.00	1.00	0.00	1.00	1.00	4.00	2.00	mod.
ID179	1.00	1.00	1.00	0.00	0.00	0.00	0.00	0.00	1.00	1.00	4.00	2.00	mod.
ID185	1.00	1.00	0.00	1.00	0.00	0.00	0.00	0.00	1.00	1.00	4.00	2.00	mod.
ID188	1.00	1.00	1.00	0.00	0.00	0.00	0.00	0.00	1.00	1.00	4.00	2.00	mod.
ID190	1.00	1.00	1.00	0.00	0.00	0.00	1.00	0.00	1.00	0.00	4.00	2.00	mod.
ID193	1.00	1.00	1.00	0.00	0.00	0.00	0.00	0.00	1.00	1.00	4.00	2.00	mod.
ID199	1.00	1.00	1.00	0.00	1.00	0.00	0.00	0.00	0.00	1.00	4.00	2.00	mod.
ID214	1.00	0.00	0.00	1.00	1.00	0.00	0.00	0.00	1.00	1.00	4.00	2.00	mod.
ID220	1.00	1.00	1.00	0.00	0.00	0.00	1.00	0.00	1.00	0.00	4.00	2.00	mod.
ID232	2.00	1.00	1.00	0.00	0.00	0.00	0.00	0.00	1.00	1.00	4.00	2.00	mod.
ID236	2.00	1.00	1.00	0.00	1.00	0.00	0.00	0.00	1.00	0.00	4.00	2.00	mod.
ID237	2.00	1.00	0.00	0.00	1.00	0.00	1.00	0.00	1.00	0.00	4.00	2.00	mod.
ID238	2.00	1.00	0.00	0.00	0.00	0.00	1.00	0.00	1.00	1.00	4.00	2.00	mod.
ID247	2.00	1.00	1.00	0.00	0.00	0.00	0.00	0.00	1.00	1.00	4.00	2.00	mod.
ID250	2.00	1.00	1.00	0.00	1.00	0.00	1.00	0.00	0.00	0.00	4.00	2.00	mod.
ID252	2.00	1.00	1.00	0.00	0.00	0.00	0.00	1.00	0.00	1.00	4.00	2.00	mod.
ID11	1.00	1.00	1.00	0.00	0.00	0.00	1.00	1.00	1.00	0.00	5.00	2.00	mod.
ID16	1.00	1.00	0.00	0.00	0.00	1.00	1.00	0.00	1.00	1.00	5.00	2.00	mod.
ID20	1.00	1.00	1.00	0.00	0.00	0.00	0.00	1.00	1.00	1.00	5.00	2.00	mod.
ID21	1.00	1.00	0.00	1.00	1.00	0.00	0.00	0.00	1.00	1.00	5.00	2.00	mod.
ID29	2.00	1.00	1.00	0.00	0.00	0.00	1.00	0.00	1.00	1.00	5.00	2.00	mod.
ID30	2.00	1.00	1.00	1.00	0.00	0.00	0.00	0.00	1.00	1.00	5.00	2.00	mod.
ID32	2.00	1.00	1.00	0.00	0.00	0.00	1.00	0.00	1.00	1.00	5.00	2.00	mod.
ID39	2.00	1.00	1.00	0.00	0.00	0.00	0.00	1.00	1.00	1.00	5.00	2.00	mod.
ID42	2.00	1.00	1.00	1.00	0.00	0.00	0.00	1.00	1.00	0.00	5.00	2.00	mod.
ID51	2.00	1.00	1.00	0.00	1.00	0.00	0.00	0.00	1.00	1.00	5.00	2.00	mod.
ID52	2.00	1.00	1.00	0.00	0.00	0.00	1.00	0.00	1.00	1.00	5.00	2.00	mod.
ID69	2.00	1.00	1.00	0.00	1.00	0.00	1.00	0.00	0.00	1.00	5.00	2.00	mod.
ID72	2.00	0.00	0.00	0.00	1.00	1.00	0.00	1.00	1.00	1.00	5.00	2.00	mod.
ID79	2.00	1.00	1.00	0.00	0.00	0.00	0.00	1.00	1.00	1.00	5.00	2.00	mod.
ID82	2.00	0.00	1.00	1.00	0.00	1.00	0.00	1.00	1.00	0.00	5.00	2.00	mod.
ID86	2.00	1.00	1.00	0.00	0.00	0.00	1.00	1.00	1.00	0.00	5.00	2.00	mod.
ID91	2.00	1.00	1.00	0.00	0.00	0.00	1.00	1.00	1.00	0.00	5.00	2.00	mod.
ID99	2.00	0.00	1.00	0.00	0.00	1.00	0.00	1.00	1.00	1.00	5.00	2.00	mod.
ID101	2.00	1.00	1.00	0.00	0.00	1.00	1.00	0.00	1.00	0.00	5.00	2.00	mod.
ID111	2.00	1.00	0.00	1.00	0.00	1.00	1.00	0.00	1.00	0.00	5.00	2.00	mod.
ID113	2.00	1.00	1.00	0.00	0.00	0.00	1.00	0.00	1.00	1.00	5.00	2.00	mod.
ID122	2.00	1.00	0.00	1.00	1.00	0.00	1.00	0.00	1.00	0.00	5.00	2.00	mod.
ID130	2.00	0.00	1.00	1.00	1.00	0.00	0.00	1.00	1.00	0.00	5.00	2.00	mod.
ID151	2.00	1.00	0.00	0.00	0.00	1.00	1.00	1.00	1.00	0.00	5.00	2.00	mod.
ID153	2.00	0.00	0.00	1.00	1.00	0.00	1.00	0.00	1.00	1.00	5.00	2.00	mod.
ID173	1.00	1.00	0.00	0.00	1.00	0.00	1.00	1.00	1.00	0.00	5.00	2.00	mod.
ID174	1.00	0.00	0.00	1.00	0.00	0.00	1.00	1.00	1.00	1.00	5.00	2.00	mod.
ID181	1.00	1.00	0.00	0.00	1.00	0.00	0.00	1.00	1.00	1.00	5.00	2.00	mod.
ID184	1.00	1.00	1.00	1.00	0.00	0.00	1.00	0.00	1.00	0.00	5.00	2.00	mod.
ID198	1.00	1.00	1.00	1.00	1.00	0.00	0.00	0.00	1.00	0.00	5.00	2.00	mod.
ID206	1.00	1.00	1.00	0.00	0.00	1.00	0.00	0.00	1.00	1.00	5.00	2.00	mod.
ID212	1.00	1.00	1.00	0.00	0.00	0.00	1.00	0.00	1.00	1.00	5.00	2.00	mod.
ID223	1.00	1.00	0.00	0.00	1.00	0.00	1.00	0.00	1.00	1.00	5.00	2.00	mod.
ID239	2.00	1.00	1.00	0.00	0.00	0.00	1.00	1.00	0.00	1.00	5.00	2.00	mod.
ID240	2.00	1.00	1.00	0.00	1.00	0.00	0.00	1.00	1.00	0.00	5.00	2.00	mod.
ID243	2.00	1.00	1.00	0.00	1.00	0.00	0.00	0.00	1.00	1.00	5.00	2.00	mod.
ID253	2.00	1.00	1.00	1.00	0.00	1.00	1.00	0.00	0.00	0.00	5.00	2.00	mod.
ID258	2.00	1.00	1.00	0.00	1.00	0.00	0.00	0.00	1.00	1.00	5.00	2.00	mod.
ID2	1.00	1.00	1.00	0.00	1.00	1.00	0.00	0.00	1.00	1.00	6.00	2.00	mod.
ID6	1.00	1.00	1.00	0.00	1.00	0.00	0.00	1.00	1.00	1.00	6.00	2.00	mod.
ID9	1.00	1.00	1.00	1.00	0.00	0.00	0.00	1.00	1.00	1.00	6.00	2.00	mod.
ID24	1.00	1.00	0.00	0.00	1.00	0.00	1.00	1.00	1.00	1.00	6.00	2.00	mod.
ID43	2.00	1.00	1.00	0.00	0.00	1.00	1.00	0.00	1.00	1.00	6.00	2.00	mod.
ID45	2.00	1.00	1.00	0.00	1.00	1.00	1.00	0.00	0.00	1.00	6.00	2.00	mod.
ID59	2.00	1.00	1.00	1.00	0.00	0.00	1.00	1.00	1.00	0.00	6.00	2.00	mod.
ID65	2.00	1.00	1.00	0.00	1.00	0.00	1.00	1.00	0.00	1.00	6.00	2.00	mod.
ID85	2.00	1.00	1.00	0.00	1.00	0.00	1.00	1.00	0.00	1.00	6.00	2.00	mod.
ID88	2.00	1.00	1.00	0.00	1.00	0.00	1.00	1.00	1.00	0.00	6.00	2.00	mod.
ID89	2.00	1.00	1.00	0.00	1.00	0.00	1.00	0.00	1.00	1.00	6.00	2.00	mod.
ID110	2.00	1.00	1.00	1.00	0.00	0.00	0.00	1.00	1.00	1.00	6.00	2.00	mod.
ID116	2.00	1.00	1.00	0.00	1.00	0.00	1.00	0.00	1.00	1.00	6.00	2.00	mod.
ID128	2.00	1.00	1.00	0.00	1.00	0.00	1.00	0.00	1.00	1.00	6.00	2.00	mod.
ID154	2.00	1.00	1.00	0.00	1.00	0.00	1.00	0.00	1.00	1.00	6.00	2.00	mod.
ID159	2.00	1.00	1.00	0.00	0.00	0.00	1.00	1.00	1.00	1.00	6.00	2.00	mod.
ID200	1.00	1.00	1.00	0.00	0.00	0.00	1.00	1.00	1.00	1.00	6.00	2.00	mod.
ID205	1.00	1.00	1.00	1.00	1.00	0.00	0.00	0.00	1.00	1.00	6.00	2.00	mod.
ID207	1.00	1.00	1.00	0.00	0.00	0.00	1.00	1.00	1.00	1.00	6.00	2.00	mod.
ID211	1.00	1.00	1.00	1.00	0.00	1.00	1.00	0.00	0.00	1.00	6.00	2.00	mod.
ID218	1.00	1.00	1.00	0.00	1.00	1.00	1.00	0.00	1.00	0.00	6.00	2.00	mod.
ID219	1.00	1.00	1.00	1.00	0.00	0.00	0.00	1.00	1.00	1.00	6.00	2.00	mod.
ID227	1.00	1.00	0.00	1.00	1.00	0.00	1.00	0.00	1.00	1.00	6.00	2.00	mod.
ID228	1.00	1.00	1.00	1.00	0.00	0.00	0.00	1.00	1.00	1.00	6.00	2.00	mod.
ID229	2.00	1.00	1.00	0.00	1.00	1.00	0.00	0.00	1.00	1.00	6.00	2.00	mod.
ID231	2.00	0.00	1.00	0.00	1.00	0.00	1.00	1.00	1.00	1.00	6.00	2.00	mod.
ID244	2.00	1.00	1.00	1.00	1.00	1.00	1.00	0.00	0.00	0.00	6.00	2.00	mod.
ID246	2.00	1.00	0.00	0.00	1.00	0.00	1.00	1.00	1.00	1.00	6.00	2.00	mod.
ID261	2.00	1.00	0.00	0.00	1.00	0.00	1.00	1.00	1.00	1.00	6.00	2.00	mod.
ID264	2.00	0.00	1.00	1.00	0.00	1.00	0.00	1.00	1.00	1.00	6.00	2.00	mod.
ID15	1.00	1.00	1.00	0.00	0.00	1.00	1.00	1.00	1.00	1.00	7.00	3.00	high
ID34	2.00	1.00	1.00	1.00	0.00	1.00	1.00	1.00	1.00	0.00	7.00	3.00	high
ID75	2.00	1.00	1.00	0.00	1.00	1.00	1.00	0.00	1.00	1.00	7.00	3.00	high
ID165	1.00	1.00	1.00	1.00	1.00	0.00	1.00	0.00	1.00	1.00	7.00	3.00	high
ID197	1.00	1.00	1.00	0.00	1.00	1.00	0.00	1.00	1.00	1.00	7.00	3.00	high
ID263	2.00	1.00	1.00	1.00	1.00	1.00	0.00	0.00	1.00	1.00	7.00	3.00	high
ID10	1.00	1.00	1.00	0.00	1.00	1.00	1.00	1.00	1.00	1.00	8.00	3.00	high
ID31	2.00	1.00	1.00	0.00	1.00	1.00	1.00	1.00	1.00	1.00	8.00	3.00	high
ID105	2.00	1.00	1.00	1.00	0.00	1.00	1.00	1.00	1.00	1.00	8.00	3.00	high
ID157	2.00	1.00	1.00	1.00	0.00	1.00	1.00	1.00	1.00	1.00	8.00	3.00	high
ID260	2.00	1.00	1.00	0.00	1.00	1.00	1.00	1.00	1.00	1.00	8.00	3.00	high
average score											3.63		

* categories: 1–3 low, 4–6 moderate, 7–9 high.

**Table A3:** Full variable importance table.

Variable	Mean Feature Importance
education level	4.779
healthy bread	3.287
fastfood	2.766
monthly household budget	2.522
work activity	2.264
mothers pre-pregnancy BMI	1.949
daily sitting pre-pregnancy	1.873
white meat	1.868
water	1.854
vegetables	1.785
milk	1.767
fruit	1.742
meat products (hot-dogs, sausages, meat pate, salami etc.)	1.715
fish	1.699
red meat	1.630
healthy muesli	1.628
coffee	1.546
pasta	1.473
sweets	1.441
tea	1.408
TV hours weekly	1.398
child weight	1.385
vegetable oil	1.353
nuts	1.328
sandwiches	1.321
pre-pregnancy weight	1.305
sauce gravy	1.291
juice	1.289
smoking in pregnancy	1.279
sugar	1.258
total children	1.242
snacks	1.222
white bread	1.204
mother height	1.147
sweetened drinks	1.134
exercise in pregnancy	1.121
number of pregnancies	1.116
yogurt	1.105
child ahead	1.085
MDSSMG score	1.056
number of household members	1.020
seafood	1.012
exercise pre-pregnancy	0.967
living sisters	0.964
dressing	0.955
unhealthy muesli	0.919
pre-pregnancy smoking	0.908
potatoes	0.888
daily sitting in pregnancy	0.868
housing status	0.862
contraception	0.851
pre-pregnancy work transportation	0.837
financial status	0.828
high activity pre-pregnancy	0.794
beans lentil	0.779
daily sleep hours	0.778
moderate activity pre-pregnancy	0.767
gestational week	0.755
dried fruit	0.750
MDSSC score	0.709
household members income	0.700
daily cigarettes household	0.695
smoking status	0.685
order of birth	0.685
moderate activity in pregnancy	0.669
work status in pregnancy	0.666
years of smoking	0.655
daily nap duration in min	0.653
light activity in pregnancy	0.628
total sons	0.627
beer	0.553
type of physical activity	0.553
working status	0.528
light activity pre-pregnancy	0.524
living brothers	0.522
work transportation in pregnancy	0.521
total daughters	0.521
quitting smoking	0.447
high activity in pregnancy	0.432
smokers in household	0.368
wine	0.368
child sex	0.343
antibiotics in pregnancy	0.337
marriages	0.334
Apgar score	0.321
infection in pregnancy	0.317
passive smoking	0.317
daily cigars pipes in household	0.315
milk substitute	0.278
pregnancy aspirin paracetamol	0.277
MDSSC cathegory	0.255
miscarriages	0.246
menstrual cycle regularity	0.224
delivery mode	0.222
high BP in pregnancy	0.211
other diseases in pregnancy	0.189
MDSSMG cathegory	0.188
previous pregnancy complications	0.164
thyroid diseases	0.157
other medication in pregnancy	0.148
inflammatory diseases in pregnancy	0.105
diabetes in pregnancy	0.095
reflux in pregnancy	0.076
deceased brothers	0.033

**Table A4:** Selected variables employed in re-training.

Variable	Mean Feature Importance
education level	6.42
healthy bread	4.62
fastfood	4.04
monthly household budget	3.51
work activity	3.06
white meat	2.85
water	2.78
pre-pregnancy BMI	2.78
vegetables	2.70
fruit	2.60
meat products (hot-dogs, sausages, meat pate, salami etc.)	2.59
daily sitting pre-pregnancy	2.59
milk	2.56
fish	2.52
red meat	2.52
healthy muesli	2.35
coffee	2.35
pasta	2.27
child weight	2.20
sweets	2.20
tea	2.15
weekly TV hours	2.12
vegetable oil	2.08
sandwiches	2.06
nuts	2.03
sauce gravy	2.01
sugar	1.97
juice	1.95
snacks	1.89
smoking in pregnancy	1.89
mothers pre-pregnancy weight	1.88
white bread	1.82
mothers height	1.78
total children	1.75
sweetened drinks	1.71
yogurt	1.70
exercise in pregnancy	1.69
MDSS MG score	1.66
seafood	1.64
child head	1.60
number of pregnancies	1.58
number of household of members	1.51
